# Synthetic investigation of competing magnetic interactions in 2D metal–chloranilate radical frameworks†

**DOI:** 10.1039/d0sc01994a

**Published:** 2020-05-11

**Authors:** Kelsey A. Collins, Richard J. Saballos, Majed S. Fataftah, Danilo Puggioni, James M. Rondinelli, Danna E. Freedman

**Affiliations:** Department of Chemistry, Northwestern University Evanston Illinois 60208 USA danna.freedman@northwestern.edu; Department of Materials Science and Engineering, Northwestern University Evanston Illinois 60208 USA jrondinelli@northwestern.edu

## Abstract

The discovery of emergent materials lies at the intersection of chemistry and condensed matter physics. Synthetic chemistry offers a pathway to create materials with the desired physical and electronic structures that support fundamentally new properties. Metal–organic frameworks are a promising platform for bottom-up chemical design of new materials, owing to their inherent chemical predictability and tunability relative to traditional solid-state materials. Herein, we describe the synthesis and magnetic characterization of a new 2,5-dihydroxy-1,4-benzoquinone based material, (NMe_2_H_2_)_3.5_Ga_2_(C_6_O_4_Cl_2_)_3_ (**1**), which features radical-based electronic spins on the sites of a kagomé lattice, a geometric lattice known to engender exotic electronic properties. Vibrational and electronic spectroscopies, in combination with magnetic susceptibility measurements, revealed **1** exhibits mixed valency between the radical-bearing trianionic and diamagnetic tetraanionic oxidation states of the ligand. This unpaired electron density on the ligand forms a partially occupied kagomé lattice where approximately 85% of the lattice sites are occupied with an *S* = ½ spin. We found that gallium mediates ferromagnetic coupling between ligand spins, creating a ferromagnetic kagomé lattice. By modulation of the interlayer spacing *via* post-synthetic cation metathesis of **1** to (NMe_4_)_3.5_Ga_2_(C_6_O_4_Cl_2_)_3_ (**2**) and (NEt_4_)_2_(NMe_4_)_1.5_Ga_2_(C_6_O_4_Cl_2_)_3_ (**3**), we determined the nature of the magnetic coupling between neighboring planes is antiferromagnetic. Additionally, we determined the role of the metal in mediating this magnetic coupling by comparison of **2** with the In^3+^ analogue, (NMe_4_)_3.5_In_2_(C_6_O_4_Cl_2_)_3_ (**4**), and we found that Ga^3+^ supports stronger superexchange coupling between ligand-based spins than In^3+^. The combination of intraplanar ferromagnetic coupling and interplanar antiferromagnetic coupling exchange interactions suggests these are promising materials to host topological phenomena.

## Abstract 2

2D metal–organic frameworks provide insight into kagomé spin physics.

## Graphical abstract

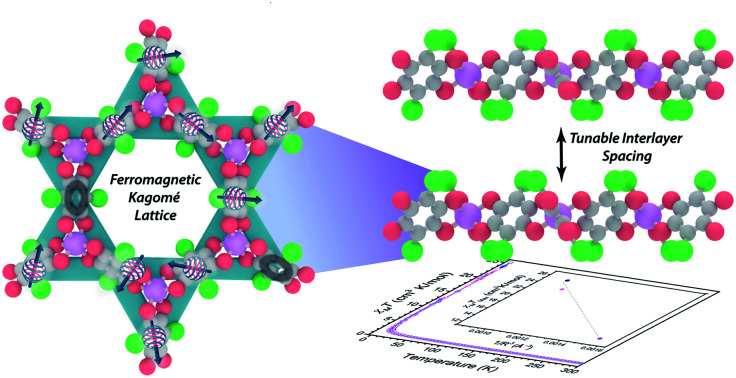

## Introduction

The discovery of new materials which host emergent phenomena lies at the intersection of condensed matter physics and synthetic chemistry. Certain lattice topologies, for example the kagomé lattice, which consists of corner sharing equilateral triangles, promote the creation of excitations which are different from those that occur in the building units of the lattice. This phenomenon is referred to as an emergent property – a property in the collective which does not occur in the core unit. Beyond emergent electronic properties, many new magnetic phases arise in spin-based materials through exchange interactions governed by the lattice geometry and the active spin and orbital degrees-of-freedom dictated by the underlying chemistry.^1^ Creating these materials from the ground up is a significant synthetic challenge which necessitates simultaneous control over both the local and extended structure and the electron filling of the frontier orbitals.

The kagomé lattice is an especially promising structure to target as either antiferromagnet or ferromagnetic interactions within the lattice lead to emergent phenomena.^1–7^ Antiferromagnetic coupling of electronic spins on the kagomé lattice leads to magnetic frustration, which arises from the competing magnetic interactions that cannot be simultaneously satisfied. This magnetic frustration prevents the onset of magnetic ordering and results in a state known as a quantum spin liquid.^1^ This state features an infinite number of degenerate magnetic ground states, and is predicted to host exotic fractionalized quasiparticles with applications in quantum computation^8^ and high temperature superconductivity.^9^ Alternatively, ferromagnetic coupling of electronic spins on a kagomé lattice leads to a different family of interesting electronic properties, and includes materials that are topological magnon band insulators,^4,5^ exhibit skyrmionic excitations,^6^ or host Dirac fermions.^7^

Two-dimensional (2D) metal–organic frameworks (MOFs) are an attractive platform for the targeted design of such new emergent materials. In contrast to traditional solid-state materials such as metal oxides and minerals, which have inherently little chemical tunability, MOFs possess a high degree of modularity as they can be built up from molecular building blocks. This modularity encompasses the identity of the metal centre, the organic bridging ligand, and the interlayer spacing. In these systems, the unpaired spin density that gives rise to exotic electronic behaviour can reside on either the metal site^6,7,10–12^ or on the organic ligand,^13–16^ expanding the number of viable synthetic targets. In these materials, the two primary components that dictate the electronic and magnetic properties are the intralayer interactions and the interlayer interactions. The former leads to the desired exotic properties of interest, while the latter often extinguishes the phenomena of interest. A prominent example is observed in antiferromagnetic kagomé materials, wherein the interlayer interactions often alleviate magnetic frustration.^17–20^ Judicious chemical design of both metal ion and organic ligand enables fine-tuning the electronic and magnetic properties of the intralayer kagomé lattice *via* a bottom-up chemical approach. Additionally, in 2D MOFs, the interlayer spacing in these materials can be modulated by intercalation of organic cations. We hypothesize that this will enable deconvolution of the magnetic behaviour as either the result of 2D interactions arising from the kagomé lattice or more complicated 3D interactions. This is an especially attractive feature of 2D MOFs over metal oxides and minerals.

**Fig. 1:**
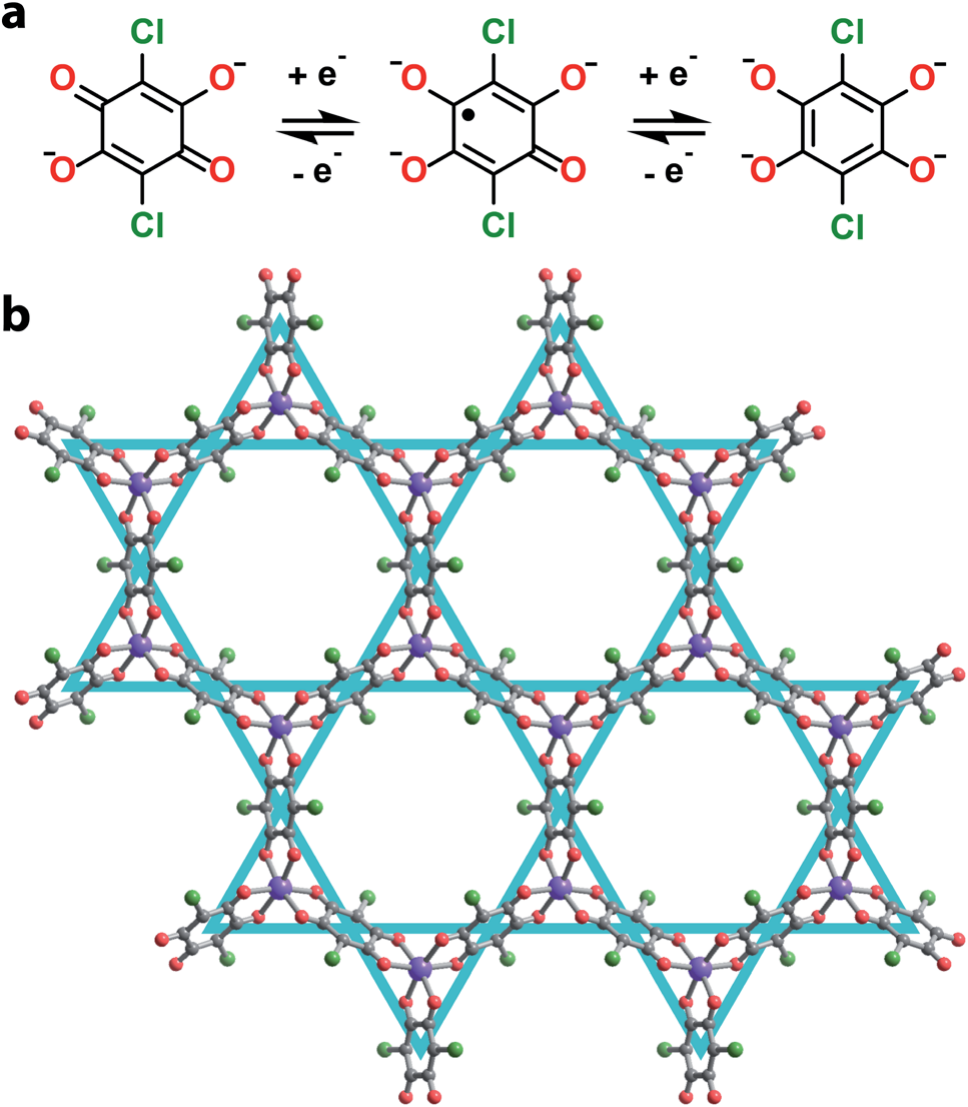
(a) Scheme of accessible oxidation states of the redox active chloranilate organic ligand. (b) Crystallographic structure of **1** determined from Rietveld refinement. Grey, red, green, and purple spheres represent carbon, oxygen, chlorine, and gallium atoms respectively. Solvent molecules and cations are omitted for clarity. The kagomé lattice is shown in teal.

Towards this end, we synthesized a series of 2D honeycomb MOFs composed of 2,5-dichloro-3,6-dihydroxy-*p*-benzoquinone (chloranilic acid) and group 13 metals (gallium and indium). In this system, chloranilate (Fig. 1a) hosts a stable organic radical in its trianionic oxidation state, localizing unpaired spin density onto the vertices of the kagomé lattice (Fig. 1b).^21^ This leads to the formation of an electronic spin based kagomé lattice on top of the structural honeycomb framework. In order to ensure the only unpaired spin density resides on the desired kagomé lattice sites, group 13 metals were chosen as the metal nodes for their diamagnetism and relative redox inertness. By preparing both the gallium and indium analogues, we are able to assess how the radial extent of the metal orbitals affect the strength of the spin–spin coupling mediated by metal–ligand superexchange. Finally, we modulate the interlayer spacing of the gallium chloranilate framework, enabling deconvolution of the inter- *vs.* intralayer magnetic interactions.

## Results and discussion

We synthesized the first 2D MOF of the series by reaction of Ga(NO_3_)_3_·*x*H_2_O with chloranilic acid in dimethylformamide (DMF) and trace amounts of water at 130 °C for 16 hours to produce green hexagonal crystals with the composition (NMe_2_H_2_)_3.5_Ga_2_CA_3_ (**1**). The structure of **1** was determined from Rietveld refinement of laboratory powder X-ray diffraction (PXRD) data. The PXRD pattern revealed the framework is isostructural with the known aluminum analogue (Fig. 1b) confirming it maintains the desired kagomé lattice structure.^22^ The Ga^3+^ ions are octahedrally coordinated by three deprotonated chloranilate ligands to form the nodes of the honeycomb net. The pores of the honeycomb are 15.56 Å in diameter and are filled with DMF solvent molecules and dimethylammonium cations formed from the decomposition of DMF during the reaction. Based upon structurally analogous frameworks and elemental analysis, these cations charge balance the anionic framework.^21–23^ The layers are eclipsed and are separated by an interlayer distance of 8.835(1) Å. The layers stack in an ABAB pattern where neighboring layers are related by a mirror plane perpendicular to the *c* axis (Fig. S5†).

**Fig. 2:**
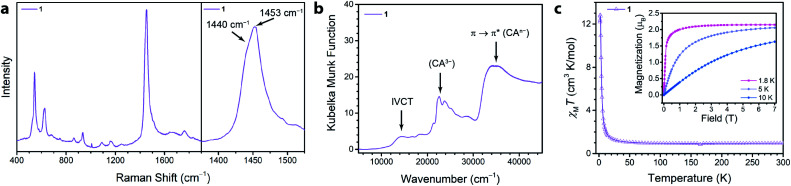
(a) Raman spectrum collected for a solid sample of **1** from 400 to 1900 cm^−1^ following excitation at 473 nm. The plot to the right highlights the main peak at 1453 cm^−1^ which has a shoulder at 1440 cm^−1^. (b) Diffuse reflectance spectrum collected for a solid sample of **1** under a N_2_ atmosphere, with arrows highlighting relevant transitions. **(c)** DC magnetic susceptibility for **1** at 0.1 T. Inset shows magnetization curves from 0 to 7 T at temperatures of 1.8, 5, and 10 K. Lines are guides to the eye.

To assess the ligand oxidation state in **1**, we performed vibrational spectroscopy as the C–C and C–O stretching modes are highly sensitive to the chloranilate ligand oxidation state.^21–24^ The frequency of the C–O stretching vibration should be largest in the CA^2−^ oxidation state and weaken as the ligand is reduced, whereas the C–C stretching vibration follows an opposite trend. After probing **1** using Raman spectroscopy, close inspection of the main band at 1453 cm^−1^ reveals an additional band at 1440 cm^−1^ (Fig. 2a). The closeness in energy of the two bands complicates assigning either as definitively to the C–C or C–O stretch. However, either band, if assigned to the C–C stretch in **1,** occurs at a much higher frequency than observed in the structural analogues (NMe_2_H_2_)_2_Zn_2_(CA^2−^)_3_ and (NMe_2_H_2_)_1.5_(CoCp_2_)_1.5_Fe_2_(CA^3^˙^−^)_3_ frameworks, which are isovalent in the dianionic and trianionic oxidation states of the ligand, respectively;^23^ the remaining band assigned as the C–O stretch is also concomitantly much weaker in **1**. Based on the observed vibrational frequencies and their near coalescence, it is evident the ligand is spontaneously reduced beyond a fully CA^3^˙^−^ system. Comparison with fully reduced chloranilic acid (H_4_CA), which displays two C–C stretches at 1448 and 1500 cm^−1^, eliminates the possibility that the bridging ligands in **1** are solely in the CA^4−^ state. Based on the aggregate of these data, we propose **1** hosts mixed valency between CA^3^˙^−^ and CA^4−^. To test our hypothesis of mixed valency, we investigated **1** by Fourier transform infrared (FTIR) spectroscopy. FTIR spectroscopy can often reveal low-lying intervalence charge transfer (IVCT) transitions in the near IR characteristic of delocalized mixed-valent species.^25^ While the FTIR spectrum of **1** did not reveal any features characteristic of mixed valency, comparison of the spectrum of **1** to molecular compounds with chloranilate in well-defined oxidation states, namely (PPh_4_)_3_[Ga(CA^2−^)_3_] and [Ga(tren)]_2_(CA^4−^)(BPh_4_)_2_ (tren = tris(2-aminoethyl)amine), which feature chloranilate exclusively in exclusively the CA^2−^ and CA^4−^ states, respectively, allows for further characterization of the ligand oxidation state. The FTIR spectrum of **1** has two intense peaks closely spaced in energy at 1403 and 1383 cm^−1^ (Fig. S6†). Both of these peaks are far weaker than would normally be assigned to the C–O double bond stretching vibration, and are much lower in energy than the C–O stretch at 1644 cm^−1^ in (PPh_4_)_3_[Ga(CA^2−^)_3_] supporting the absence of CA^2−^. Additionally, the peak at 1383 cm^−1^ is considerably lower in energy than the C–C stretching mode in [Ga(tren)]_2_(CA^4−^)(BPh_4_)_2_ (1467 cm^−1^). However, in the case of mixed valency of CA^3^˙^−^ and CA^4−^, there should only be one or two formal C–O double bonds per every three ligands. This change in bonding should significantly weaken (and strengthen) the C–O (and C–C) stretching vibrational mode. The aggregate of this data further supports our assignment of mixed-valent ligand oxidation states in **1** (Table 1).

**Table 1:** Summary of C–O and C–C bond frequencies of **1** and molecular comparison species as determined with Raman spectroscopy

Compound	*ν* (C–O) (cm^−1^)	*ν* (C–C) (cm^−1^)
**1**	1437	1450
(NMe_2_H_2_)_1.5_(CoCp_2_)_1.5_Fe_2_(CA^3^˙^−^)_3_ (ref. 22)	1487	1390
(NMe_2_H_2_)_2_Zn_2_(CA^2−^)_3_ (ref. 22)	1617	1360
H_4_CA	—	1448, 1500

The absence of an IVCT band by FTIR spectroscopy motivated us to examine electronic absorption spectroscopy to further probe the ligand mixed valency in these frameworks. The diffuse reflectance data (Fig. 2b) collected for **1** featured many transitions across the energy range of inspection (7500–45 000 cm^−1^). The peak at ∼21 000 cm^−1^ was assigned as the π* → π* transition of CA^3^˙^−^ and features a fine structure associated with the C–O vibrational modes which has been previously observed in molecular species containing CA^3^˙^−^.^26,27^ The broad, intense electronic transition at 35 000 cm^−1^ is analogous to the π → π* transition of 1,2-dihydroxybenzoquinone and was likewise assigned to the same transition in chloranilate.^22,26^

Of more immediate interest is the presence of a broad, low-intensity peak at 14 400 cm^−1^ which is tentatively assigned as an IVCT band. Due to the broadness, weak intensity, and relatively high energy of the peak, we assigned **1** as a weakly exchanging Class II Robin–Day mixed-valent material.^28^ Similar low intensity features in the near IR were also observed in a structurally similar chromium(iii) chloranilate framework and were assigned to an IVCT consistent with a localized electronic structure.^29^ Conversely, strongly exchanging Class II and Class III Robin–Day mixed-valent chloranilate frameworks have lower energy and more intense IVCT bands.^29,30^ The presence of an IVCT band in **1** is in contrast to mononuclear homoleptic gallium(iii) complexes that are mixed-valent in the ligand oxidation state but lack an IVCT band in their electronic spectra.^31,32^ In these complexes, gallium(iii) is a poor bridging metal ion and does not facilitate strong electronic communication between the two oxidation states of the ligands. This suggests that inter-ligand electronic communication is stronger in the framework than in corresponding molecular complexes. To test these assignments of the electronic transitions, we exposed **1** to air, leading to oxidation of the CA^3^˙^−^ ligands to their CA^2−^ forms. Indeed, the ligands in **1** are oxidized to CA^2−^, and as a result, the peaks assigned to CA^3^˙^−^ and the IVCT disappear, concurrent with the appearance of a peak at 18 700 cm^−1^, which is assigned to the π → π* transition of CA^2−^ (see Fig. S13 and S14 and ESI for extended discussion†).

To quantify the degree of mixed valency in **1**, we investigated its magnetic properties using SQUID magnetometry. Specifically, variable-temperature dc magnetic susceptibility measurements allow us to directly assess the number of unpaired spins in the framework by quantitation of the high-temperature *χ*_M_*T* value, thus elucidating the ratio of CA^3^˙^−^ to CA^4−^ in **1**. The 300 K *χ*_M_*T* value of **1** is 0.96 cm^3^ K mol^−1^, which persists down to 100 K (Fig. 2c). This value is below the *χ*_M_*T* value of 1.125 cm^3^ K mol^−1^ expected for 3 uncoupled radical spins but above the *χ*_M_*T* value of 0.750 cm^3^ K mol^−1^ expected for 2 uncoupled radical spins. This *χ*_M_*T* value corresponds to approximately 83% of the ligands being in the CA^3^˙^−^ state, and 17% in the CA^4−^, and further supports the ligand mixed valency suggested by our analysis of the vibrational and diffuse-reflectance data.

Below 100 K, *χ*_M_*T* rises and reaches a maximum value of 12.82 cm^3^ K mol^−1^ at 2 K. This rise in *χ*_M_*T* is attributed to ferromagnetic coupling between the ligand-based spins that does not however lead to magnetic ordering. To support the presence of significant ferromagnetic coupling in **1**, we measured the magnetization of **1**. At 1.8 K, the magnetization rapidly saturates (Fig. 2C, inset). By 0.15 T, the magnetization curve is no longer linear with field, and by 0.50 T the material is fully saturated. We hypothesize the ferromagnetic coupling arises from intralayer coupling of spins within the same 2-D layer of the framework, as intralayer interactions are expected to be much stronger than interlayer interactions. This type of ferromagnetic interligand coupling was also observed in molecular complexes of Ga(iii) tris–semiquinone complexes.^31,33,34^ It was proposed that the empty 4p orbitals of Ga(iii) mediated a ferromagnetic superexchange interaction between the radical bearing π* orbitals of the semiquinone ligands.^33^ This pathway could also be responsible for the ferromagnetic coupling in **1**.

As noted above, competing magnetic interactions arising from interlayer and intralayer magnetic coupling often have dramatic effects on any potential exotic electronic behavior hosted by these materials. The synthetic modularity of this 2D framework yields an opportunity to investigate the nature of the interlayer magnetic coupling, and deconvolute it from intralayer magnetic interactions. We can probe the relative magnitude and nature of the interlayer coupling by modulation of the interlayer spacing by post-synthetic cation exchange. Based on literature reports of anilate MOFs synthesized with different alkyl ammonium cations without a distortion of the 2D lattice, we pursued the intercalation of bulky quaternary alkyl ammonium cations to expand the interlayer spacing without distorting the kagomé lattice.^35,36^

**Fig. 3:**
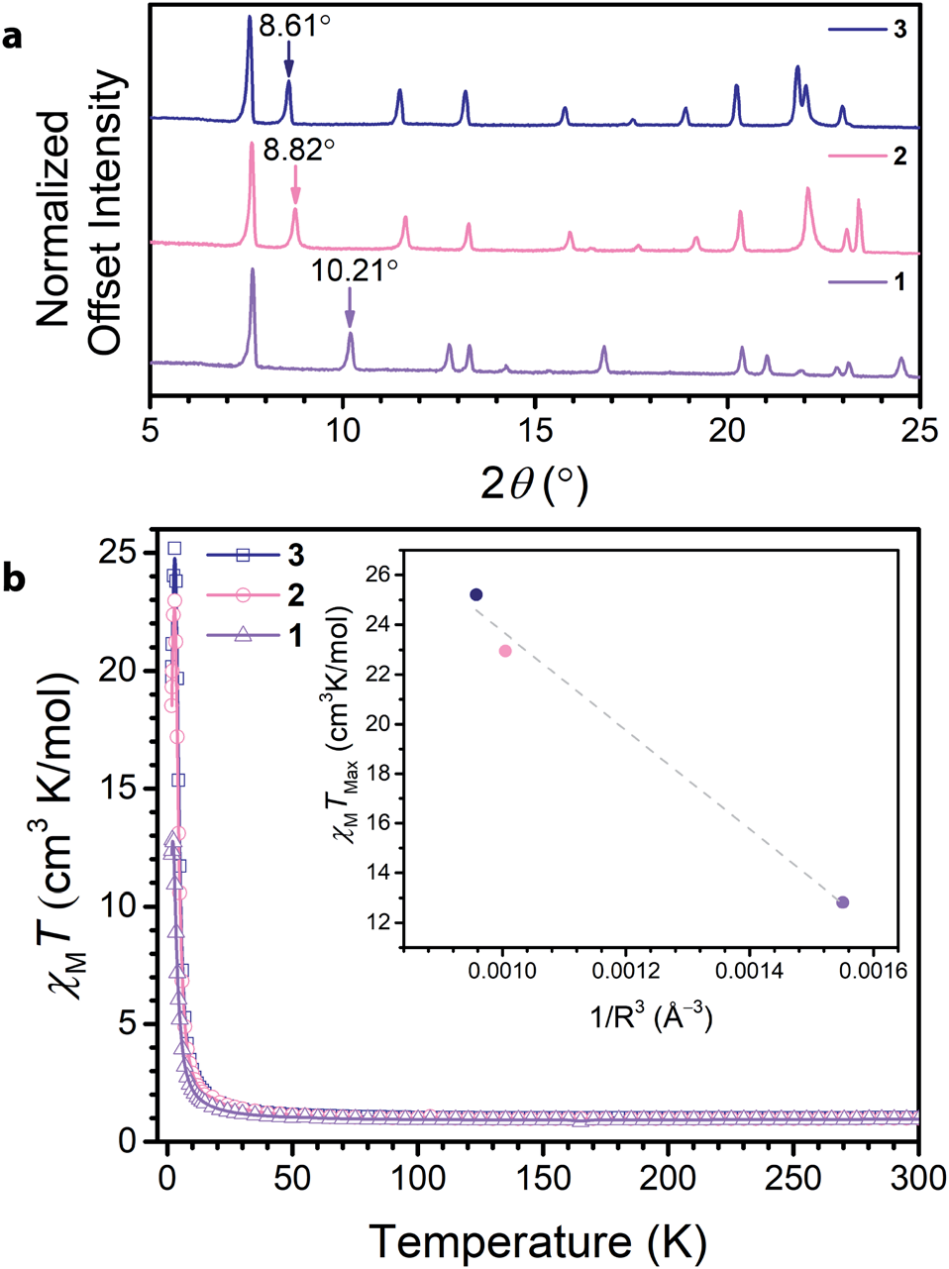
(a) PXRD of **1**, **2**, and **3** collected with Cu Kα_1_ radiation showing the expansion of the interlayer spacing with intercalation of tetraalkylammonium cations. The arrows point to the (002) peak of each pattern that corresponds to the interlayer spacing and is modulated by the size of the cation. (b) DC magnetic susceptibility of **1**, **2**, and **3**, displaying the dependence of the low temperature magnetic behaviour on the interlayer spacing. The inset highlights the dependence of *χ*_M_*T*_max_ on 1/*R*^3^, where *R* is the interlayer spacing. The data are fit to a linear relationship with an *R*^2^ of 0.981, demonstrating the strong correlation between the maximum magnetic moment and the interlayer spacing.

Soaking **1** in a 0.15 M solution of NMe_4_OH in DMF at 75 °C for 12 hours led to the isolation of (NMe_4_)_3.5_Ga_2_CA_3_ (**2**). Subsequently, soaking **2** in a 0.1 M solution of tetraethylammonium bromide in DMF at 75 °C for 12 hours resulted in the formation and isolation of (NEt_4_)_2_(NMe_4_)_1.5_Ga_2_CA_3_ (**3**). Comparison of the PXRD data across the series revealed a clear dependence of the (002) peak on cation metathesis, with a shift in the (002) peak from 10.21° to 8.82° to 8.61° in moving from **1** to **3** (Fig. 3a). These shifts in 2*θ* point to changes in the interlayer spacing across the series, ranging from 8.835(1) Å to 10.06(2) Å to 10.155(4) Å, from **1** to **3**, respectively. These data suggest that as in isostructural frameworks with transition metal nodes, the NR_4_^+^ cations reside between the Ga^3+^ centres of neighbouring layers and act to modulate the interlayer spacing.^35,36^ When this interlayer site is fully occupied, these sites account for two of the cations per formula unit; by elemental analysis, the remaining one and a half negative charges from the framework are charge balanced by tetramethylammonium ions we believe reside in the pore of the framework. Importantly, the incorporation of NR_4_^+^ cations does not distort the 2D framework as the only affected peaks observed by PXRD are those associated with the *c*-axis, corresponding to the interlayer direction. Additionally, this cation metathesis process does not affect the ligand oxidation state, as the relevant vibrational modes observed by Raman and FTIR spectroscopy remain unchanged (see ESI Fig. S8–S10†).

To further probe the ligand oxidation states of **2** and **3**, and to evaluate their magnetic properties, we collected variable-temperature dc magnetic susceptibility data. The 300 K magnetic moments measured for **2** and **3** resemble that for **1**, further corroborating that post-synthetic cation exchange does not affect ligand oxidation state (Fig. 3b). Close inspection of the low-temperature (<50 K) *χ*_M_*T* data reveals a divergence, leading to different maximum values of *χ*_M_*T* at 3 K. To contrast the interlayer magnetic interactions across the series, the maximum *χ*_M_*T* value (*χ*_M_*T*_max_) observed at low temperatures serves as a proxy for the overall strength of the ferromagnetic coupling in the material. The observed *χ*_M_*T*_max_ for **1** of 12.82 cm^3^ K mol^−1^ dramatically increases upon interlayer expansion of 1.7 Å, reaching a *χ*_M_*T*_max_ of 22.96 cm^3^ K mol^−1^ in **2**. Separating the layers further in **3** leads to a modest increase in *χ*_M_*T*_max_ to 25.20 cm^3^ K mol^−1^. For antiferromagnetic coupling between layers, *χ*_M_*T*_max_ should increase upon interlayer separation, as the magnetic coupling between layers weakens. Indeed, upon expansion of the interlayer spacing across the series, *χ*_M_*T*_max_ concomitantly rises, suggesting the presence of antiferromagnetic interlayer coupling. Though the enhancement of *χ*_M_*T*_max_ is more modest between **2** and **3** than it is from **1** to **2**, it is important to note this change arises from an increased layer separation of only 0.10 Å. Interestingly, the inset of Fig. 3b reveals *χ*_M_*T*_max_ varies relatively linearly with decreasing 1/*R*^3^ where *R* is the layer separation, in the compounds. While the possibility of cation interaction cannot be excluded, these data demonstrate a clear trend with increasing layer separation. This observation supports the presence of antiferromagnetic dipolar coupling between the 2D kagomé layers.^37,38^

We also sought to investigate the role of the diamagnetic metal in mediating radical–radical coupling by synthesizing the indium(iii) analogue. However, the direct reaction of indium(iii) nitrate hydrate and chloranilic acid in DMF and water leads to the formation of (NMe_2_H_2_)_4_In_2_(CA^3^˙^−^)_2_(CA^4−^), which hosts a different percentage of ligands in the radical state than **1** (see ESI for details†). In order to circumvent this obstacle, we targeted a tetramethylammonium based indium framework *via* direct reaction of the starting materials. Reaction of In(NO_3_)_3_·*x*H_2_O, chloranilic acid, and 5 equivalents of NMe_4_OH in DMF produces (NMe_4_)_3.5_In_2_(CA)_3_ (**4**). Unlike (NMe_2_H_2_)_4_In_2_(CA^3^˙^−^)_2_(CA^4−^), **4** contains the same percentage ligands in their radical form as **1–3**, allowing direct comparison of the magnetic behaviour of **2** and **4**. The high temperature DC magnetic susceptibility data show the radical-bearing ligands are in the same percentage in **2** and **4** (see ESI†). At low temperatures, the magnetic behavior greatly differs, and *χ*_M_*T*_max_ is much greater for **2** than **4**; the difference in *χ*_M_*T*_max_ is 15.5 cm^3^ K mol^−1^.

We next performed electronic structure calculations to elucidate the microscopic origin of the enhanced spin–spin interactions (higher *χ*_M_*T*_max_) in the gallium compounds. Our density functional theory (DFT) calculations were performed using the Vienna *Ab initio* Software Package (VASP) with the projected augmented wave method.^39,40^ The aforementioned compounds were modelled using the experimental structures as initial atomic configurations without solvent molecules, *i.e.*, as Ga_2_CA_3_ (*P*6_3_/*mcm* symmetry) and In_2_CA_3_ (*P*3̄).^41^ We achieved a CA^3^˙^−^ (spin ½) configuration by electron doping the chloranilate anions and then imposing ferromagnetic spin alignment among these ligands within the 2D kagomé layers, which couple antiferromagnetically. The internal atomic positions of each structure was relaxed using the experimental volume.

First, we found that the diamagnetic metal–oxygen bond lengths from the bidentate CA^3^˙^−^ ligands were shorter for Ga_2_CA_3_ (1.98 Å) compared to In_2_CA_3_ (2.16 Å), consistent with the smaller ionic radius of Ga^3+^ (187 pm) compared to In^3+^ (220 pm). This leads to larger orbital overlap and enhanced charge density in the bonding region (indicated in Fig. S19†), which initiates the superexchange path between sites, Ga–O–C–C–C–O–Ga; although the *S* = ½ spin state is distributed about the chloranilate anion, it is predominately localized on the oxygen anions. In addition, the GaO_6_ octahedral units are closer to ideal compared to the InO_6_ owing to deviations of the intra-octahedral oxygen–metal–oxygen angles from 90°, which narrow the electronic bandwidth. As a result, the direct distance between coupled chlorinate anions and cations is significantly shorter in Ga_2_CA_3_ (3.84 Å) compared to In_2_CA_3_ (4.049 Å). Second, analysis of the electronic density-of-states reveals that in both compounds, the valence band is mainly comprised of the chloranilate anion states with chlorine 3p orbitals located below (from −5 to −3.5 eV) the oxygen 2p states (spanning −3 to −1.5 eV), whereas the conduction band consists of the main group s and p states. Although the empty gallium states are located at higher energy than those of indium (Fig. S19†), they show a small overlap with the occupied oxygen 2p bands. This mixing is stronger for gallium compared to indium over the −4 to −2 eV energy range (Fig. S19†). Furthermore, the Ga compound exhibits greater p-orbital occupancy (0.35e) than In (0.31e), demonstrating that there is stronger hybridization about the p states than s states (0.31e, 0.32e; Fig. S20†).

We therefore concluded that chelated diamagnetic metals favor ferromagnetic spin–spin coupling, owing to orthogonal symmetry of the p orbitals from the metal and oxide ligands consistent with trends reported for iminobenzosemiquinonato group 13 molecular complexes.^34,42^ Moreover, because stronger spin–spin interactions arise when there are stronger hybridization (covalent interactions) among the active orbitals participating in superexchange, this coupling is greater for gallium owing to the greater exchange propagated by the orbital hybridization. Indeed, upon approximating the exchange interaction by finding the energy difference between the FM and AFM configurations (where a C ring has an opposite spin with respect to the other two rings), we found that energy difference was larger for Ga (40 K) compared to In (17 K).

The observation of topological behaviour necessitates that each kagomé lattice site hosts a full *S* = ½ spin, leading us to pursue post-synthetic chemical oxidation of the ligand to achieve a purely CA^3−^ framework. Previous work has demonstrated that honeycomb-type MOFs of chloranilate can successfully undergo single-crystal to single-crystal post-synthetic chemical reduction from a mixed-valent CA^2−/3^˙^−^ system to a fully CA^3^˙^−^ system.^23^ Thus motivated, we sought to treat the framework with an oxidant with the ability to oxidise CA^4−^ to CA^3^˙^−^, without effecting the oxidation of CA^3^˙^−^ to CA^2−^. Soaking **1** in a solution of ferrocenium hexafluorophosphate in acetonitrile results in oxidation of the CA^4−^ ligand to CA^3^˙^−^, as evidenced by vibrational and electronic spectroscopies. Peaks in the Raman spectrum grow in at 1390 and 1505 cm^−1^ (Fig. S29†), which are consistent with the C–C and C–O stretches previously observed for CA^3^˙^−^.^23^ However, a remnant Raman peak at 1445 cm^−1^, suggests incomplete oxidation of the CA^4−^ ligand to the CA^3^˙^−^ based framework. The IR peaks in **1** at 1405 and 1381 cm^−1^ undergo significant shifts to 1481 and 1361 cm^−1^ (Fig. S30†), which is attributed to a strengthening of the C–O bond and weakening of the C–C bond, respectively, as the percentage of CA^3^˙^−^ in the framework increases. Further, the oxidation of CA^4−^ to CA^3^˙^−^ goes to completion as evidenced by the disappearance of the IVCT band at 14 500 cm^−1^ after post-synthetic oxidation (Fig. S31†). Accurate quantitation of the magnetic properties of the oxidized material is precluded by the presence of excess ferrocenium ions in the pores of the framework. Ongoing work is focused on post-synthetic oxidation of **1–3** to the fully CA^3^˙^−^-based framework with the exclusion of paramagnetic cations convoluting our interpretation of the magnetic properties.

## Conclusions

The bottom-up design of 2D materials with the potential to display exotic, emergent properties is an emerging challenge for synthetic chemists. Creating a pathway to realize these materials is a crucial step forward in an area dominated by traditional solid-state chemistry. The foregoing results demonstrate the synthesis of a series of 2D honeycomb MOFs using molecular building blocks geared towards accessing a kagomé lattice of unpaired electronic spins. The synthetic modularity of these 2D frameworks enables clear deconvolution of the inter- *vs.* intralayer magnetic interactions allowing the confirmation of ferromagnetic coupling within the kagomé plane and antiferromagnetic coupling between kagomé layers. Our data enabled us to establish the form of magnetic coupling within the layer and between the layers, thereby enabling us to assign intraplane ferromagnetic coupling interactions. The observation and confirmation of ferromagnetic coupling interactions within a kagomé plane suggests this may be a candidate for future study, in particular with the fully radical-bearing end member of the series.

## Conflicts of interest

There are no conflicts to declare.

## Supplementary Material

SC-011-D0SC01994A-s001

SC-011-D0SC01994A-s002
