# Supplementary material for: Synthetic investigation of competing magnetic interactions in 2D metal–chloranilate radical frameworks
Source: Chem Sci. 2020 May 11;11(23):5922–8. doi: 10.1039/d0sc01994a (PMC8159288; doi:10.1039/d0sc01994a)
Supplement: SC-011-D0SC01994A-s001 [file SC-011-D0SC01994A-s001.pdf]

Electronic Supplementary Material (ESI) for Chemical Science.
This journal is © The Royal Society of Chemistry 2020

Electronic Supplementary Information for:

Synthetic Investigation of Competing Magnetic Interactions in 2D Metal-Organic Frameworks

Kelsey A. Collins,[1] Richard Saballos,[2] Majed S. Fataftah,[1] Danilo Puggioni,[2] James Rondinelli,[2]* and Danna E. Freedman[1]*

[1] *Department of Chemistry, Northwestern University, Evanston, Illinois 60208.*

[2] *Department of Materials Science and Engineering, Northwestern University, Evanston, Illinois 60208.*

Table of Contents

**Full experimental details**	**S3–7**
**Discussion of ligand oxidation state**	**S6–7**
**Discussion of spin variability**	**S7**
**Difference in oxidation state between 1 and $(NMe_2H_2)_4In_2CA_3$**	**S7**
**Discussion of elemental analysis and MOF solvation**	**S8**
**Table S1 |** Crystallographic information for the structural refinement of $(NMe_2H_2)_4In_2CA_3$.	**S9**
**Table S2 |** Unit Cell Parameters of **1 – 4**	**S10**
**Figures S1 – S4 |** PXRD and fit of **1 – 4**	**S10–13**
**Figure S5** | Crystal structure of **1** along crystallographic *b* axis	**S14**
**Figure S6** | Crystal structure of $(NMe_2H_2)_4In_2CA_3$	**S15**
**Figure S7** | Crystal structure of $(NMe_2H_2)_4In_2CA_3$ along crystallographic *b* axis	**S16**
**Figures S8 – S9 |** Resonance Raman spectra of **1 – 4**	**S17**
**Figure S10 |** FTIR spectra of **1 – 4**	**S18**
**Figure S11 |** FTIR spectra of **1** and model complexes	**S19**
**Figure S12 |** FTIR spectra of **1** and **1·air**	**S20**
**Figure S13 |** Diffuse reflectance spectra of **1** and **1·air**	**S21**
**Figure S14 |** Diffuse reflectance spectra of **1 – 4**	**S22**
**Figure S15 |** FTIR spectra of **1** and $(NMe_2H_2)_4In_2CA_3$	**S23**
**Figure S16 |** Resonance Raman spectra of **1** and $(NMe_2H_2)_4In_2CA_3$	**S24**
**Figure S17 |** Diffuse reflectance spectrum of $(NMe_2H_2)_4In_2CA_3$	**S25**
**Figure S18 |** DC magnetic susceptibility comparison of **2** and **4**	**S26**
**Figures S19 |** Calculated charge density and projected density of states of Ga_2CA_3 and In_2CA_3	**S26**
**Figures S20 |** Integrated density of states for Ga_2CA_3 and In_2CA_3	**S27**
**Figures S21 – S24 |** Variable field magnetization of **1 – 4**	**S28–29**
**Figures S25 – S28 |** 100 K magnetization of **1 – 4**	**S30–31**
**Figure S29 |** Resonance Raman spectra of **1** and **1·ox**	**S32**
**Figure S30 |** FTIR spectra of **1** and **1·ox**	**S32**
**Figure S31 |** Diffuse reflectance spectra of **1** and **1·ox**	**S33**

Full Experimental Details

General considerations: All manipulations and synthetic procedures were performed under a N_2 atmosphere with either an MBraun Unilab Pro glovebox, Vacuum Atmosphere Nexus II glovebox, or Schlenk techniques. Glassware was either oven-dried at 150 °C for at least four hours and/or flame-dried prior to use. Acetonitrile (MeCN), methanol (MeOH), and diethylether (Et_2O), and dimethylformamide (DMF) were dried using a commercial solvent purification system from Pure Process Technology. All other reagents were used as received. $Ga_2(tren)_2(C_6O_4Cl_2)(BPh_4)_2$ was prepared according to literature methods.[1]

$(NMe_2H_2)_{3.5}Ga_2(C_6O_4Cl_2)_3{\cdot}1H_2O{\cdot}4.6DMF$ (1): In a dinitrogen atmosphere in an MBraun LABstar glovebox, operated under a humid atmosphere, $Ga(NO_3)_3{\bullet}7.67\ H_2O$ (0.5947 g, 1.513 mmol) and chloranilic acid (0.6229 g, 2.982 mmol) were combined in DMF (80 mL) with H_2O (1 mL) in 100 mL jar with a Teflon lined lid to give a purple suspension. This mixture was transferred to a 130 °C oven for 16 hours to afford a green microcrystalline powder. The reaction was transferred back into the same MBraun glovebox, and then filtered and washed with DMF (2 × 50 mL), yielding 96.8 mg. IR (cm^{-1}): 2926, 2507, 2159, 1668, 1553, 1500, 1403, 1383, 1256, 1211, 1090, 1042, 1027, 886, 849, 746, 659, 577, 522, 434; see Figure S7 for full spectrum. Combustion analyses calculated for (found): 36.56 (36.59) %C; 4.91 (4.97) %H; 8.91 (8.85) %N.

$(NMe_4)_{3.5}Ga_2(C_6O_4Cl_2)_3{\cdot}1.6H_2O{\cdot}4.6DMF$ (2): In a dinitrogen atmosphere in an MBraun LABstar glovebox, operated under a humid atmosphere, **1** was soaked in 10 mL of a 0.15 M solution of tetramethylammonium hydroxide pentahydrate in DMF at 75°C for 12 hours to yield $(NMe_4)_{3.5}Ga_2(C_6O_4Cl_2)$ (**2**). The reaction was filtered and washed with DMF (2 × 10 mL). IR (cm^{-1}): 3027, 2924, 2858, 1661, 1482, 1405, 1384, 1254, 1192, 1090, 1042, 1007, 949, 840, 825, 745, 657; see Figure S7 for full spectrum. Combustion analyses calculated for (found): 39.72 (39.69) %C; 5.65 (5.53) %H; 8.21 (8.28) %N.

$(NEt_4)_2(NMe_4)_{1.5}Ga_2(C_6O_4Cl_2)_3{\cdot}1H_2O{\cdot}5.8DMF$ (3): In a dinitrogen atmosphere in an MBraun LABstar glovebox, operated under a humid atmosphere, **2** was soaked in 10 mL of a 0.15 M solution of tetraethylammonium bromide in DMF at 75°C for 12 hours to yield $(NEt_4)_{3.5}Ga_2(C_6O_4Cl_2)$ (**3**). The reaction was filtered and washed with DMF (2 × 10 mL). IR (cm^{-1}):

2981, 2921, 2853, 1664, 1557, 1404, 1384, 1257, 1176, 1091, 1040, 1002, 844, 786, 658, 604, 574, 547, 434; see Figure S7 for full spectrum. Combustion analyses calculated for (found): 43.80 (39.69) %C; 6.44 (5.53) %H; 8.28 (8.28) %N.

$(NMe_2H_2)_4In_2CA_3{\cdot}6.9H_2O{\cdot}4.1DMF$: In a dinitrogen atmosphere in an MBraun LABstar glovebox, operated under a humid atmosphere, $In(NO_3)_3{\bullet}5\ H_2O$ (79.0 mg, 0.212 mmol) and chloranilic acid (68.3 mg, 0.327 mmol) were combined in DMF (10 mL) with water (1 mL) in a 22 mL vial with a Teflon lined lid to give a purple solution. This reaction was transferred to a 130 °C oven for 16 hours to afford a green crystalline material. The reaction was transferred back into the same MBraun glovebox, and then filtered and washed with DMF (2 × 10 mL) yielding 29.3 mg. IR (cm^{-1}): 3087, 3030, 2798, 1551, 1530, 1408, 1379, 1274, 1262, 1197, 1024, 961, 875, 846, 741, 675, 533, 506, 436. Combustion analyses calculated for (found): 31.55 (31.36) %C; 5.15 (4.74) %H; 7.78 (8.24) %N.

$(NMe_4)_{3.5}In_2(C_6O_4Cl_2)_3{\cdot}1.7H_2O{\cdot}5.5DMF$ (4): In a dinitrogen atmosphere in an MBraun LABstar glovebox, operated under a humid atmosphere, $In(NO_3)_3{\bullet}5\ H_2O$ (0.337 g, 0.964 mmol), chloranilic acid (0.325 g, 1.56 mmol), and tetramethylammonium hydroxide pentahydrate (0.700 g, 3.87 mmol) were combined in DMF (70 mL) in a 100 mL jar with a Teflon lined lid to give a purple suspension. This mixture was transferred to a 130 °C oven for 16 hours to afford a green microcrystalline powder. The reaction was transferred back into the same MBraun glovebox, and then filtered and washed with DMF (2 × 50 mL), yielding 100 mg. IR (cm^{-1}): 2924, 2853, 1666, 1539, 1482, 1399, 1386, 1257, 1089, 1020, 948, 839, 658; see Figure S7 for full spectrum. Combustion analyses calculated for (found): 37.78 (37.73) %C; 5.48 (5.33) %H; 8.18 (8.29) %N.

$(PPh_4)_3Ga(C_6O_4Cl_2)_3$: The molecular complex was prepared according to modified literature procedure for $(NEt_3H)_3In(C_6O_4Cl_2)_3$.[2] $GaCl_3$ (78.6 mg, 0.446 mmol) and chloranilic acid (386.7 mg, 1.842 mmol) were combined in methanol (10 mL) and stirred. Hünig's base (361.4 mg, 2.796 mmol) was added dropwise to the solution and stirred for 2 hours. Ph_4PBr (582.2 mg, 1.389 mmol) was added to the solution to precipitate a red-violet solid. The solid was dissolved in acetonitrile and crystallized from a vapor diffusion of diethyl ether into the solution of the complex. Diffuse reflectance; cm^{-1} (F(R)): 19,195 (7.455), 27,742 (12.247), 31,761 (21.506), 36,354 (19.180),

37,340 (19.306), 44,067 (17.739). IR (cm^{-1}): 3055, 3019, 1644, 1518, 1435, 1347, 1299, 1187, 1163, 1104, 995, 839, 753, 720, 687, 599, 570, 523; see Figure S8 for full spectrum.

Raman Measurements: Raman spectra were collected using a Horiba LabRam HR Evolution confocal microscope. Samples were excited with a 473 nm continuous-wave diode laser (Cobalt Blue) equipped with a long working distance 50× microscope objective and 600 grooves/mm grating. The resolution of the instrument was 3 cm^{-1}. Spectra were collected for 5 seconds each and 12 spectra were averaged per sample.

Magnetic Susceptibility Measurements: Direct-current (dc) susceptibility measurements were performed with Quantum Design MPMS-XL SQUID magnetometer. Samples of **1**–**4** were prepared under an inert atmosphere as finely ground microcrystalline powders sealed in quartz tubes. All samples were restrained with eicosane to prevent crystallite torqueing and ensure good thermal contact with the thermal bath. Dc susceptibility measurements were performed at fields of 1000, 5000, 10000 Oe. The data were corrected for the diamagnetic contributions of the sample holder and the sample itself through the use of Pascal's constants.[3] Magnetization curves (*M* vs. *H*) were recorded at 100 K, from 0–4 T to check for the presence of unwanted ferromagnetic impurities.

Powder X-Ray Diffraction: Frameworks were transferred either to a boron-rich capillary tube with 0.7 mm outer diameter or sandwiched between pieces of Kapton tape in a nitrogen-filled humid glovebox. The capillary tube was sealed with UV curable epoxy and illuminated with a UV flashlight for 5 minutes before removal from the glovebox. Powder X-ray diffraction patterns were collected on a STOE STADI MP diffractometer equipped with CuKα1 (compound **4**) or MoKα1 (compounds **1** – **3**) radiation.

X-ray Structure Determination. All diffraction data were collected in the X-ray crystallography lab of the Integrated Molecular Structure Education and Research Center (IMSERC) at Northwestern University. Single crystals of $(NMe_2H_2)_3In_2CA_3$ were coated in deoxygenated Paratone-N oil and mounted on a MicroMounts rod. The crystallographic data were collected at 100 K on a Rigaku XtaLAB Synergy, Single source at home/near, HyPix diffractometer equipped

with a Cu Kα PhotonJet X-ray source. Raw data were integrated and corrected for Lorentz and polarization effects using CrysAlisPro 1.171.40.53 (Rigaku OD, 2019). The space group assignment was determined by examination of systematic absences, E-statistics, and successive refinement of the structure. The structure was solved and refined with SHELXL operated with the OLEX2 interface. Thermal parameters were refined anisotropically for all atoms.

Other Physical Measurements: Combustion analyses were performed by Midwest Microlab Inc. (Indianapolis, IN). Infrared spectra were recorded on a Bruker Alpha FTIR spectrometer equipped with an attenuated total reflectance accessory and diamond anvil with a resolution of $4 cm^{-1}$. UV-Vis spectra were collected with a Varian Cary 5000 spectrophotometer with a Praying Mantis diffuse reflectance attachment.

Computational Details: Density functional theory was carried out using the Vienna ab inito software package[4] (VASP) with the Projected augmented wave[5,6] (PAW) basis set. The pseudopotentials used are as follows: C $2s^2 2p^2$, O $2s^2 2p^4$, Cl $3s^2 3p^5$, Ga $3d^{10} 4s^2 4p^1$, In $4d^{10} 5s^2 5p^1$. The calculations use the PBEsol functional.[7] A kinetic energy cutoff of 600 eV was used in conjunction with a 4x4x3 Monkhorst-Pack *k*-point mesh[8] to relax the atomic positions to have forces less than 10^{-5} eV $Å^{-1}$.

Discussion of ligand oxidation state determined with FTIR: The two strongest peaks in **1** are at 1403 and 1380 cm^{-1} (the peak at 1650 cm^{-1} is from DMF molecules in the pores of the framework) and are assigned to the CC and CO stretches of chloranilate in the framework respectively (see Figure S8). The CO stretch is much weaker than what is observed in $(PPh_4)_3Ga(CA^{2-})_3$, where chloranilate is exclusively dianionic, indicating that spontaneous reduction of the chloranilate ligand occurs during reaction of gallium (III) nitrate and chloranilic acid in DMF. The CC stretch is also weaker than what is observed in $Ga_2tren_2CA^{4-}(BPh_4)_2$ indicating that the reduction of chloranilate does not go to completion and that instead there is a mix of CA^{3-} and CA^{4-} in **1** (Figure S8). When **1** is exposed to air, the CO (CC) stretch strengthens (weakens) to 1520 cm^{-1} (1350 cm^{-1}) as would be expected for oxidizing a mixture of CA^{3-} and CA^{4-} to CA^{2-} (Figure S9).

Discussion of ligand oxidation determined with diffuse reflectance spectroscopy: After air exposure, the ligand is oxidized from the mixture of the CA^{3-} and CA^{4-} oxidation states to the CA^{2-} oxidation state as evidenced by the decrease in the $\pi^* \rightarrow \pi^*$ transition of CA^{3-} at 21,000 cm^{-1}, the growth of the peak at 19,000 cm^{-1} consistent with CA^{2-}, and the decrease of the weak IVCT band at 14,500 cm^{-1} (Figure S10). The disappearance of the peak at 14,500 cm^{-1} concomitant with the growth of the CA^{2-} peak CA^{2-} at 19,000 cm^{-1} supports the assignment of the former peak as an IVCT band.

Discussion of spin variability: Different batches of **1** had a small degree of variation in the exact ratio of CA^{3-} and CA^{4-}, as evidenced by the high temperature (> 100 K) DC SQUID magnetometry. The high temperature magnetic moments ranged from 0.85 to 0.95 cm^3K/mol, indicating a variation in CA^{3-} percentage from 75% to 84%.In order to avoid this variation in radical population convoluting the comparison of **1** – **3**, the post-synthetic cation treatments and subsequent magnetic analysis were done on the same initial batch of **1**.

Difference in oxidation state between 1 and $(NMe_2H_2)_4In_2CA_3$: Attempting to isolate the isostructural and isoelectronic indium framework of **1** via the direct synthesis outlined above results in $(NMe_2H_2)_4In_2CA_3$ where statistically only two of the three chloranilate ligands in the unit cell have radical character; the ratio of the ligand is 2 CA^{3-}:1 CA^{4-}. This difference in oxidation state was determined via FTIR and resonance Raman spectroscopies (Figures S12 and S13). The dominant stretches in the FTIR spectrum of $(NMe_2H_2)_4In_2CA_3$ are at 1409 and 1378 cm^{-1}; though this may seem like a small difference compared to the stretches of **1** at 1403 and 1383 cm^{-1}, this does support $(NMe_2H_2)_4In_2CA_3$ having a slightly stronger (weaker) C-C (C-O) stretch than **1**. This difference in CA^{3-}:CA^{4-} ratio between frameworks is further corroborated by the resonance Raman spectra, where the difference in bond energies is even more apparent. Whereas **1** has a Raman peak at 1453 cm^{-1} with a shoulder at 1440 cm^{-1}, $(NMe_2H_2)_4In_2CA_3$ has two clearly resolved peaks at 1437 and 1469 cm^{-1}, indicative of a stronger (weaker) C-C (C-O) stretch in agreement with the conclusions from the FTIR analysis. The strengthening of the C-C stretch concomitant with the weakening of the C-O stretch supports a more reduced ligand oxidation state in $(NMe_2H_2)_4In_2CA_3$. These two spectroscopic techniques definitively show **1** and $(NMe_2H_2)_4In_2CA_3$ have different ratios of CA^{3-} and CA^{4-}.

Discussion of elemental analysis and MOF solvation: In order to ensure that we fully understood the stoichiometry of the cations and solvation of the sample we used for magnetic analysis, elemental analysis was collected on a portion of the same batch that was used for magnetic analysis. The stoichiometry of the dimethylammonium cation was fixed, and residual carbon, hydrogen, and nitrogen abundance was assigned to DMF and H_2O solvent molecules in the MOF.

Table S1. Crystallographic information for the structural refinement of $(NMe_2H_2)_4In_2CA_3$.

Empirical Formula	$C_9Cl_3InO_6$
Formula weight	425.24 g/mol
Temperature	100.0(1) K
Wavelength	1.54181 Å
Crystal System	Trigonal
Space Group	P−31m
Unit Cell Dimensions	a = 13.899(14) Å, □ = 90°
	b = 13.899(14) Å, □ = 90°
	c =8.292(4) Å, □ = 120°
Volume	1372(3) Å^3
Z	2.00004
Density (calculated)	1.029 g/cm^3
Absorption coefficient	9.671 mm^{-1}
F_{000}	404.0
Crystal color	Green
Crystal size	0.02 × 0.05 × 0.07 mm^3
2ϑ range	7.344 to 105.526°
Index ranges	$-12 \leq h \leq 7$
	$-12 \leq k \leq 14$
	$-4 \leq l \leq 8$
Reflections collected	2119
Independent reflections	550 [R_{int} = 0.0931]
Completeness to □ = 101°	100 %
Absorption correction	Gaussian
Maximum and minimum transmission	0.802 and 0.639
Refinement method	Full-matrix least-squares on F^2
Data / restraints / parameters	550/20/33
Goodness-of-fit on F^{2a}	1.097
Final R indices [I > 2□(I)]^b	R_1 = 12.53 %, wR_2 = 32.52 %
R indices (all data, 0.80 Å)	R_1 = 18.17 %, wR_2 = 37.76 %
Largest diff. peak and hole	0.68 and –0.54 e.Å^{-3}

^{a} GooF = $[\Sigma[w(F_o^2 - F_c^2)^2] / (n-p)]^{1/2}$ where n is the number of reflections and p is the total number of parameters refined. $^b R_1 = \Sigma||F_o| - |F_c|| / \Sigma|F_o|$; $wR_2 = [\Sigma[w(F_o^2 - F_c^2)^2] / \Sigma[w(F_o^2)^2]]^{1/2}$

Table S2 | Unit cell parameters for **1** – **4** obtained from Rietveld (**1**) or Pawley (**2-4**) refinement of powder x-ray diffraction data.

	**Space Group**	***a* (Å)**	***c* (Å)**	**Volume (Å^3)**
**$(NMe_2H_2)_{1.5}Al_2CA_3$**	$P6_3/mcm$	13.13487(84)	17.4947(11)	2613.89(37)
**1**	$P6_3/mcm$	13.480(4)	17.669(2)	2780.5(1.3)
**2**	$P6_3/mcm$	13.41(1)	20.12(1)	3132.2(4.6)
**3**	$P6_3/mcm$	13.386(4)	20.310(4)	3151.6(1.7)
**4**	$P6_3/mcm$	14.011(6)	19.861(4)	3376.8(2.2)

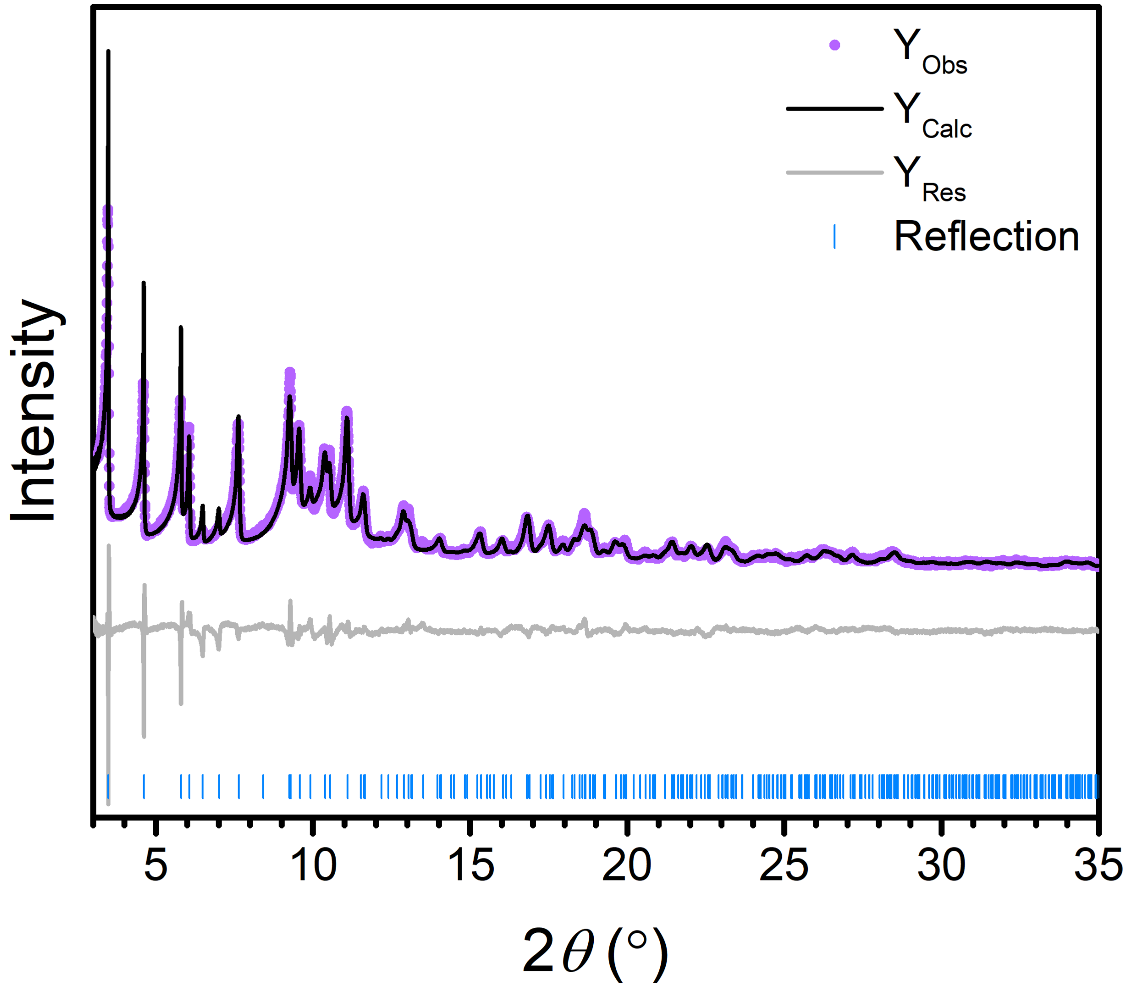

Figure S1. Powder x-ray diffraction data for **1**. Data were collected at room temperature with pure Mo Kα1 radiation. R_{wp} = 4.376 %. Y_{obs}, Y_{calc}, Y_{res}, and Reflection are the observed, calculated, and residual reflection intensity and the predicted reflections, respectively.

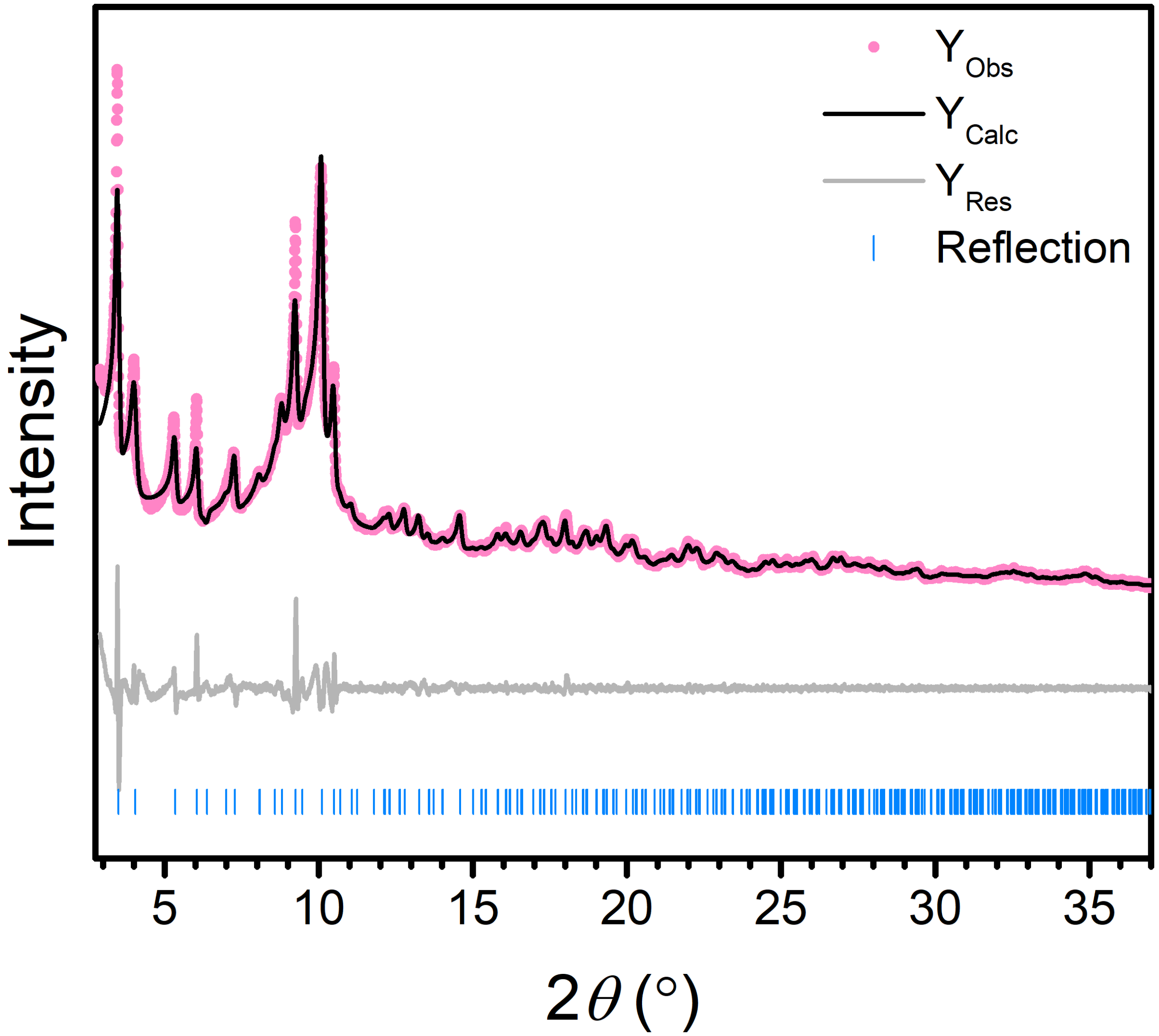

Figure S2 | Powder x-ray diffraction data for **2**. Data were collected at room temperature with pure Mo Kα1 radiation. R_{wp} = 4.010%. Y_{obs}, Y_{calc}, Y_{res}, and Reflection are the observed, calculated, and residual reflection intensity and the predicted reflections, respectively.

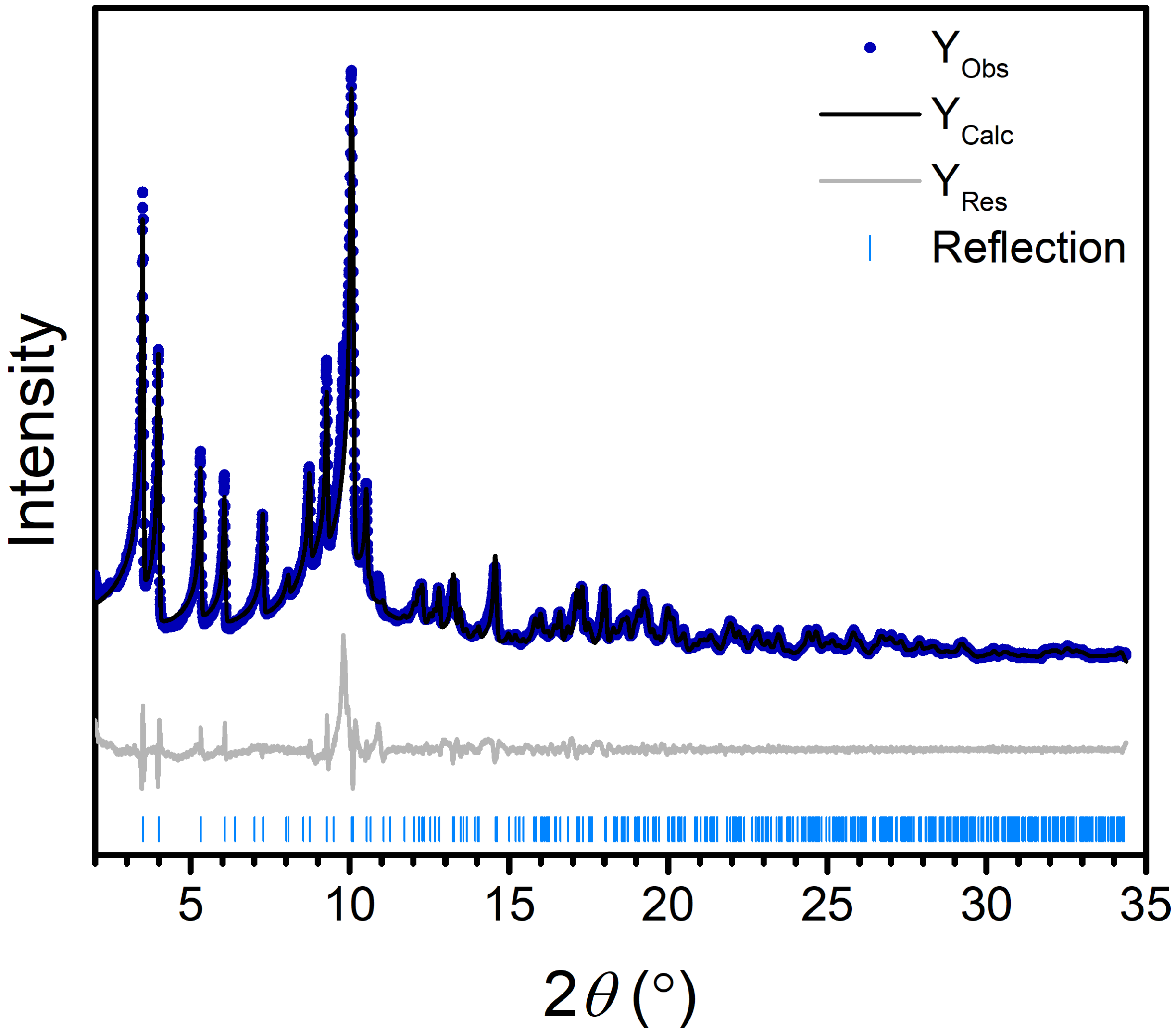

Figure S3 | Powder x-ray diffraction data for **3**. Data were collected at room temperature with pure Mo Kα1 radiation. R_{wp} = 3.128 %. Y_{obs}, Y_{calc}, Y_{res}, and Reflection are the observed, calculated, and residual reflection intensity and the predicted reflections, respectively.

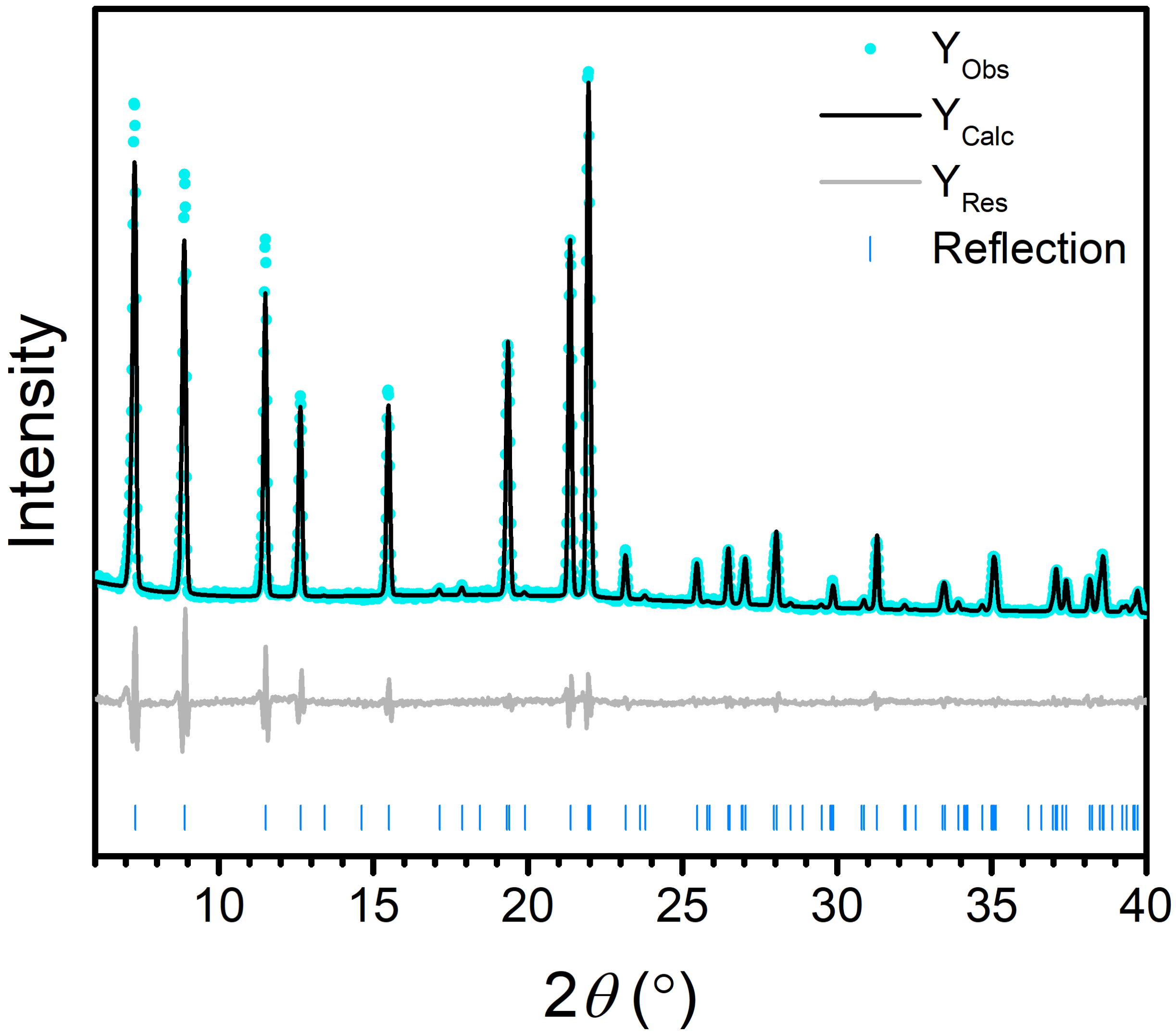

Figure S4 | Powder x-ray diffraction data for **4**. Data were collected at room temperature with pure Cu Kα1 radiation. R_{wp} = 9.562%. Y_{obs}, Y_{calc}, Y_{res}, and Reflection are the observed, calculated, and residual reflection intensity and the predicted reflections, respectively.

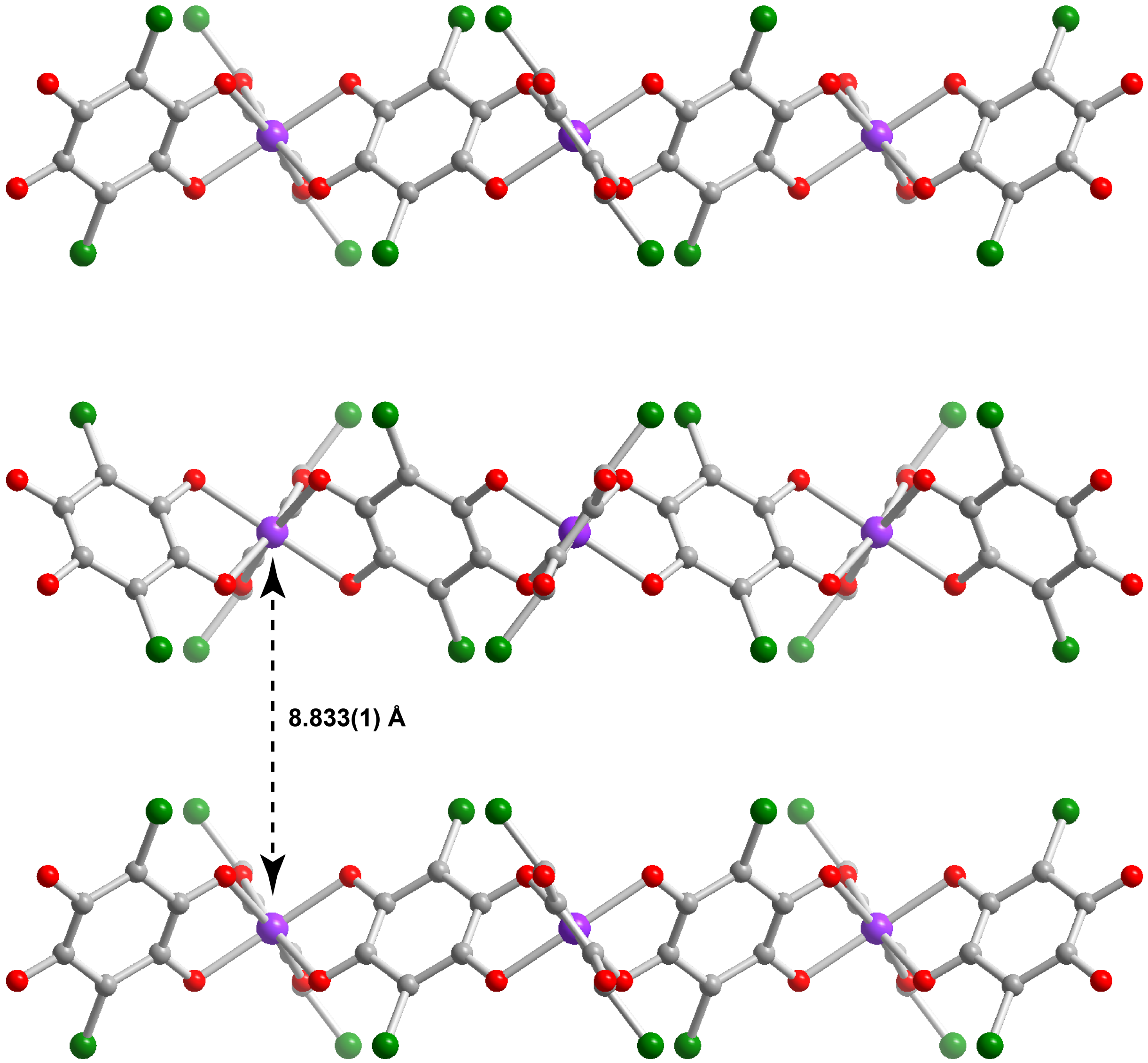

Figure S5 | Crystal structure of **1** viewed along the crystallographic *b* axis, showing the interlayer distance of 8.833(1) Å, which is half of the crystallographic *c* axis of the unit cell. Solvent molecules are omitted for clarity. Purple, grey, red, and green spheres are gallium, carbon, oxygen, and chlorine atoms, respectively.

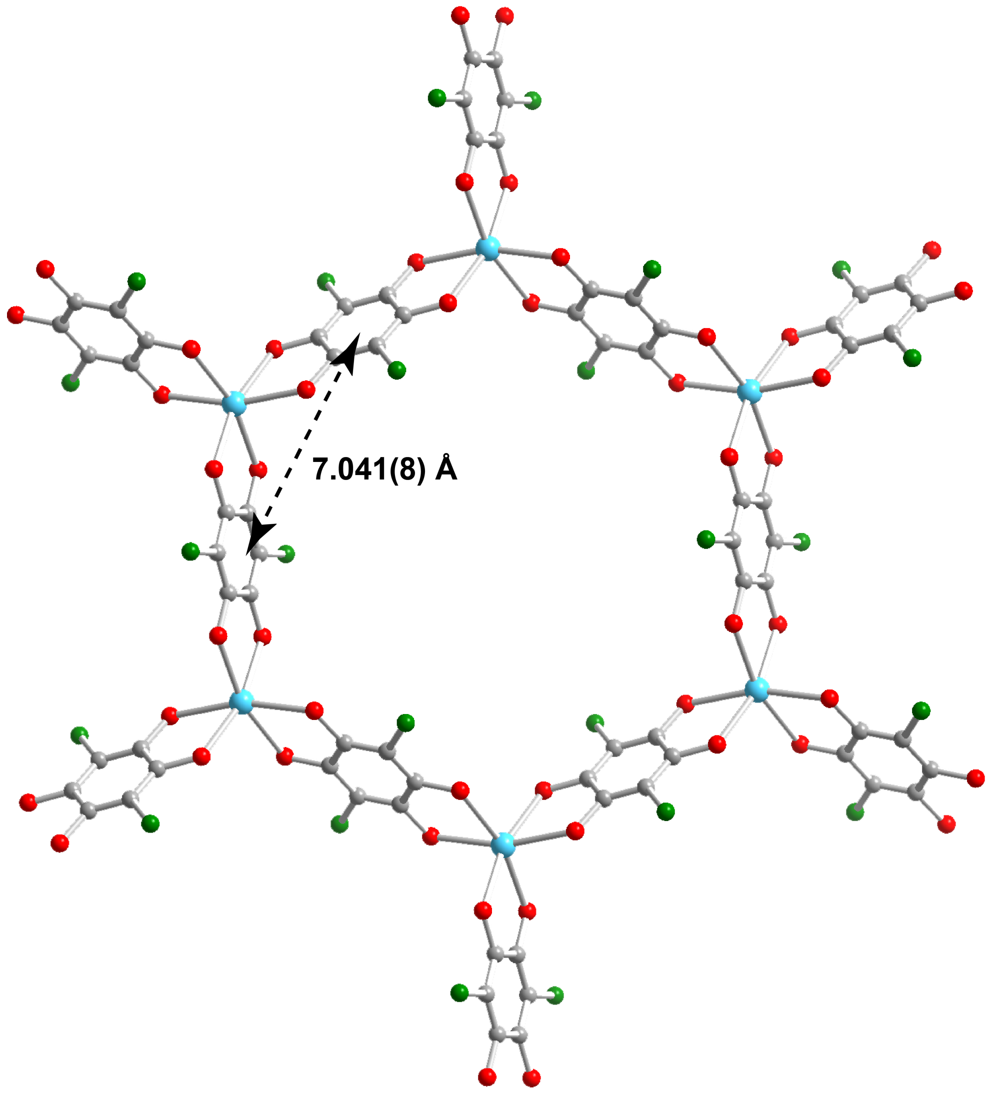

Figure S6 | Crystal structure of $(NMe_2H_2)_4In_2CA_3$ viewed along the crystallographic *c* axis, showing the spin-spin distance of 7.041(8) Å, which is calculated as the distance between the rings of neighbouring chloranilate ligands. In **1**, this distance is 6.8397 Å. Cyan, grey, red, and green spheres are indium, carbon, oxygen, and chlorine atoms, respectively.

.

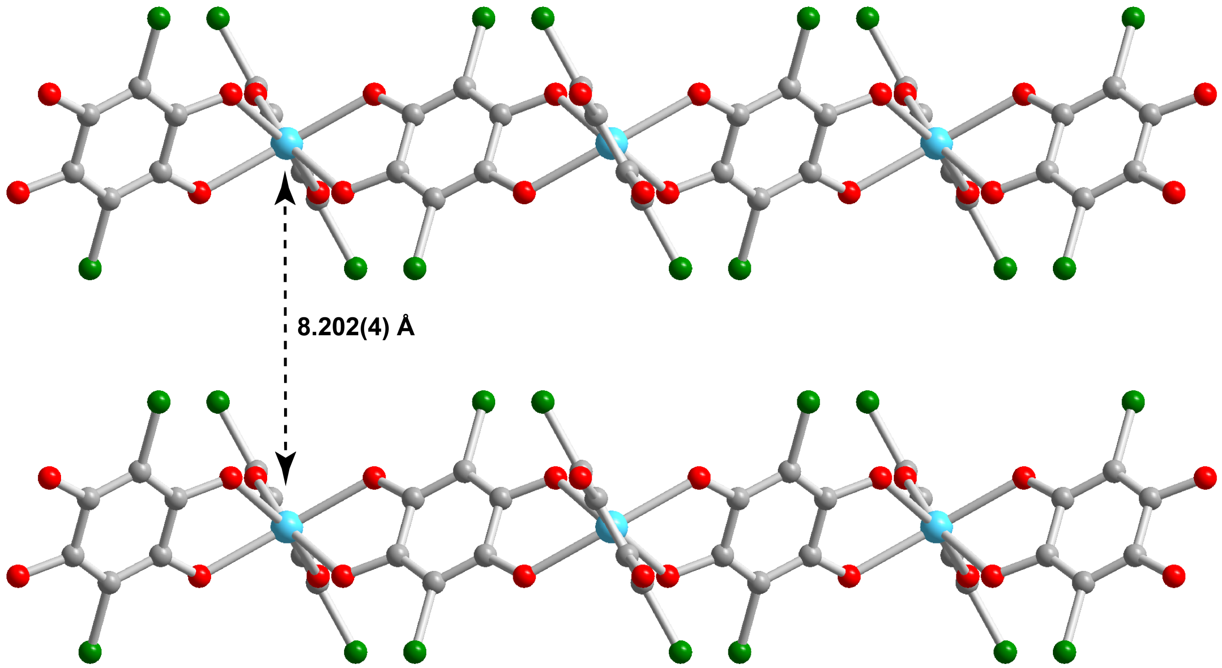

Figure S7 | Crystal structure of $(NMe_2H_2)_4In_2CA_3$ viewed along the crystallographic *b* axis, showing the interlayer distance of 8.202(4) Å. Cyan, grey, red, and green spheres are indium, carbon, oxygen, and chlorine atoms, respectively.

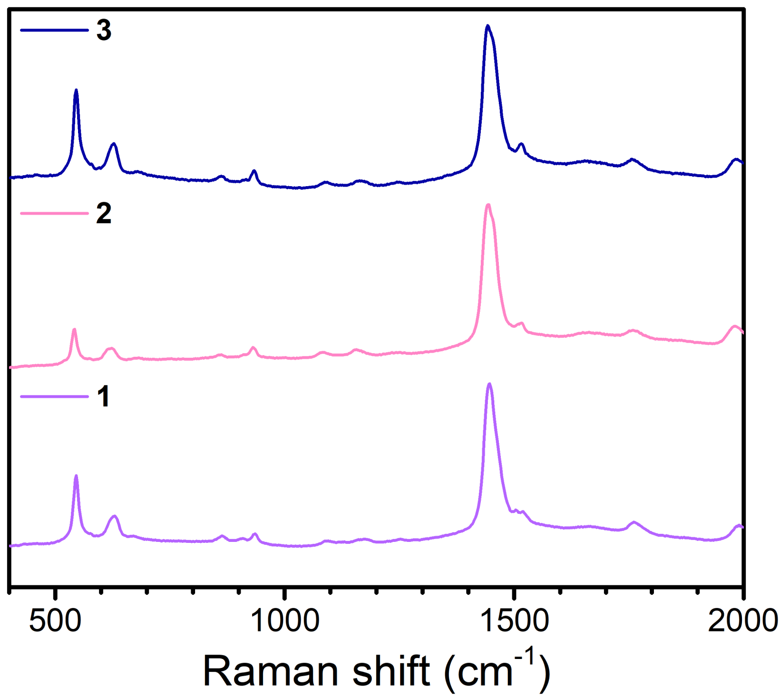

Figure S8 | Resonance Raman spectroscopic data on **1**, **2**, and **3**. All data were collected in sealed boron-rich glass capillaries at room temperature with λ_{ex} = 473 nm.

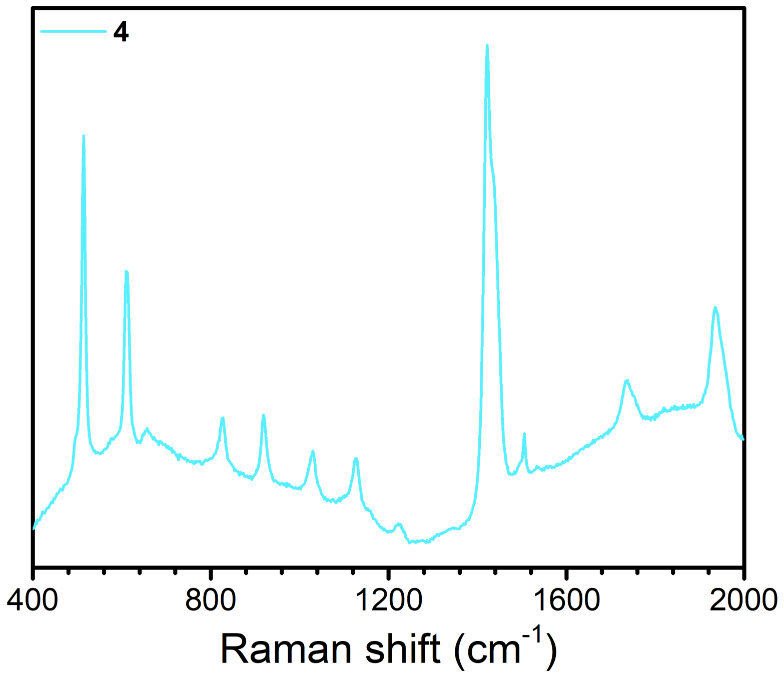

Figure S9 | Resonance Raman spectroscopic data on **4**. All data were collected in sealed boron-rich glass capillaries at room temperature with λ_{ex} = 473 nm.

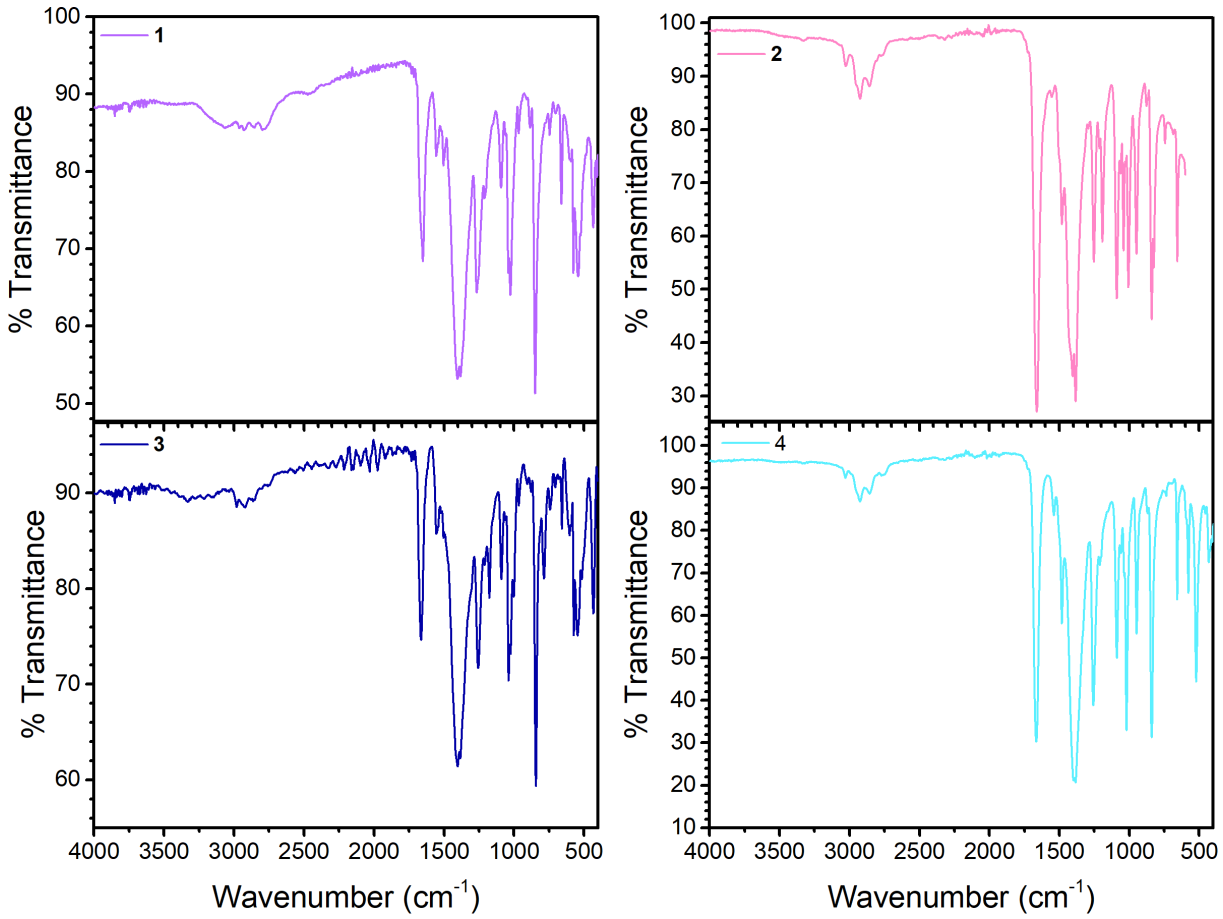

Figure S10 | Infrared spectroscopic data on **1**–**4**. All data were collected on solid samples at room temperature under a dinitrogen atmosphere.

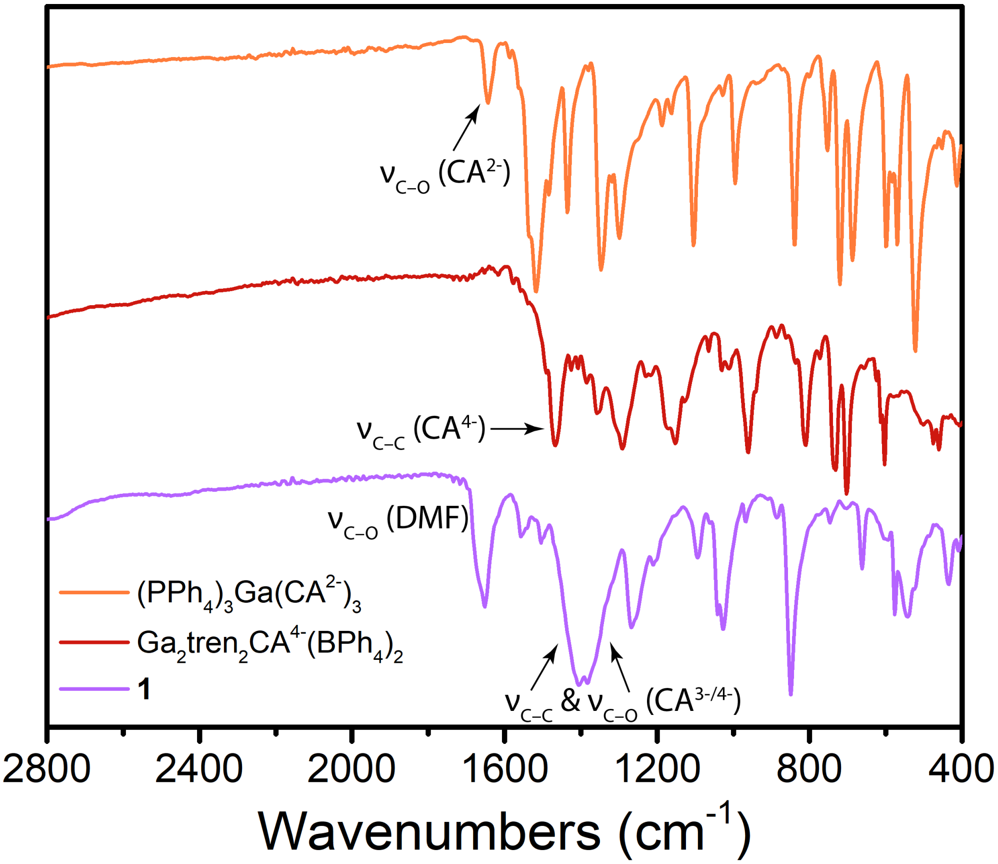

Figure S11 | Stacked infrared spectra of **1** (purple), $(PPh_4)_3Ga(CA^{2-})_3$ (orange), and $Ga_2tren_2CA^{4-}(BPh_4)_2$ (red). All data were collected on solid samples at room temperature under a dinitrogen atmosphere. The relevant C-C and C-O stretches are highlighted for comparison.

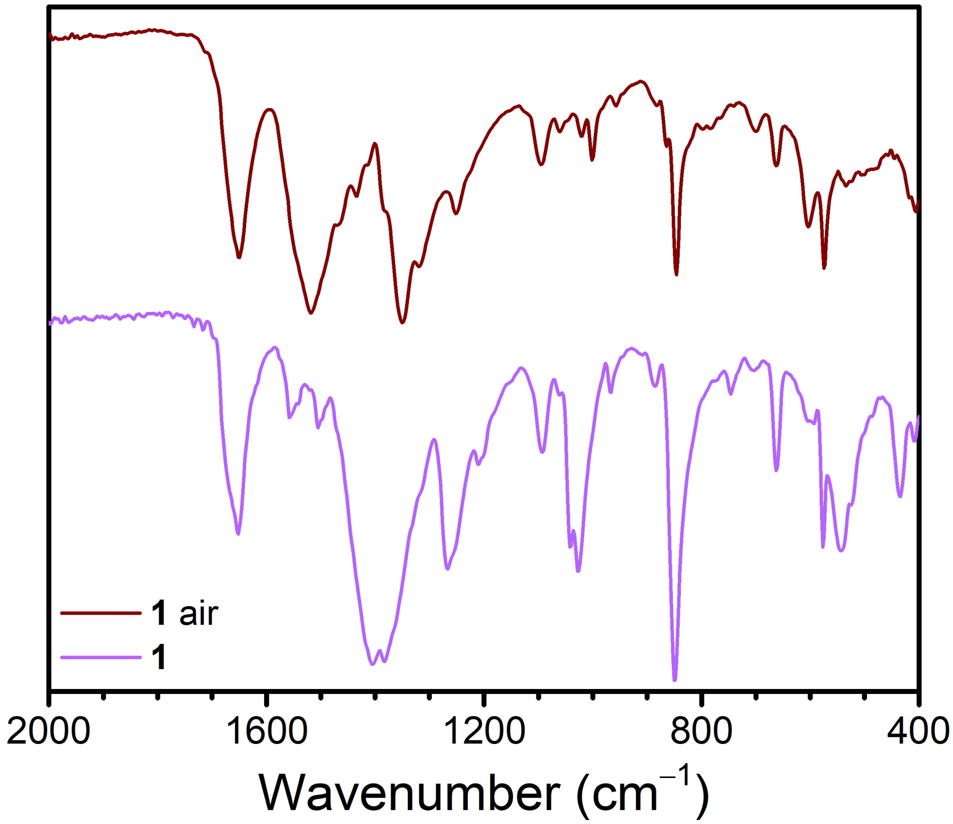

Figure S12 | Infrared spectroscopic data on **1** (purple) and **1** that was exposed to air (brown). All data were collected on solid samples at room temperature under a dinitrogen atmosphere for **1** and under ambient atmosphere for **1** air.

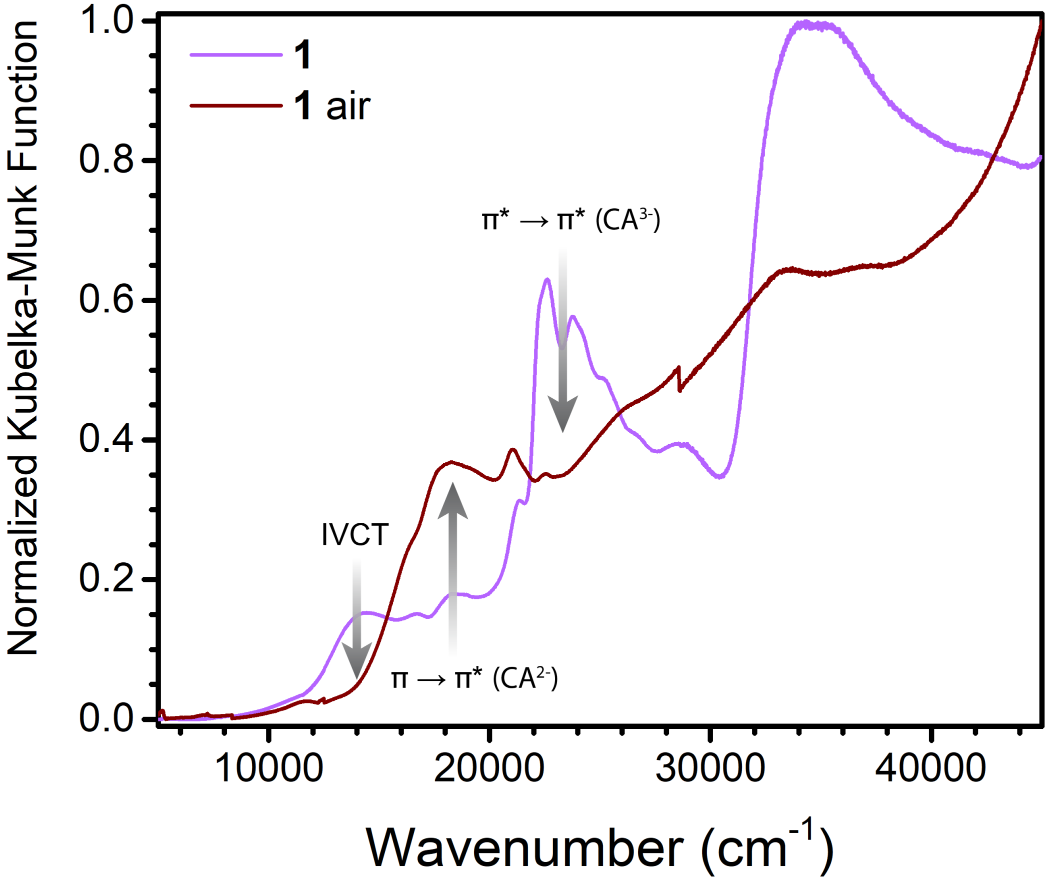

Figure S13 | Diffuse reflectance (UV-Vis) spectra for **1** and a sample of **1** that was open to air for 48 hours**.** Spectra were collected at room temperature under nitrogen and air, respectively.

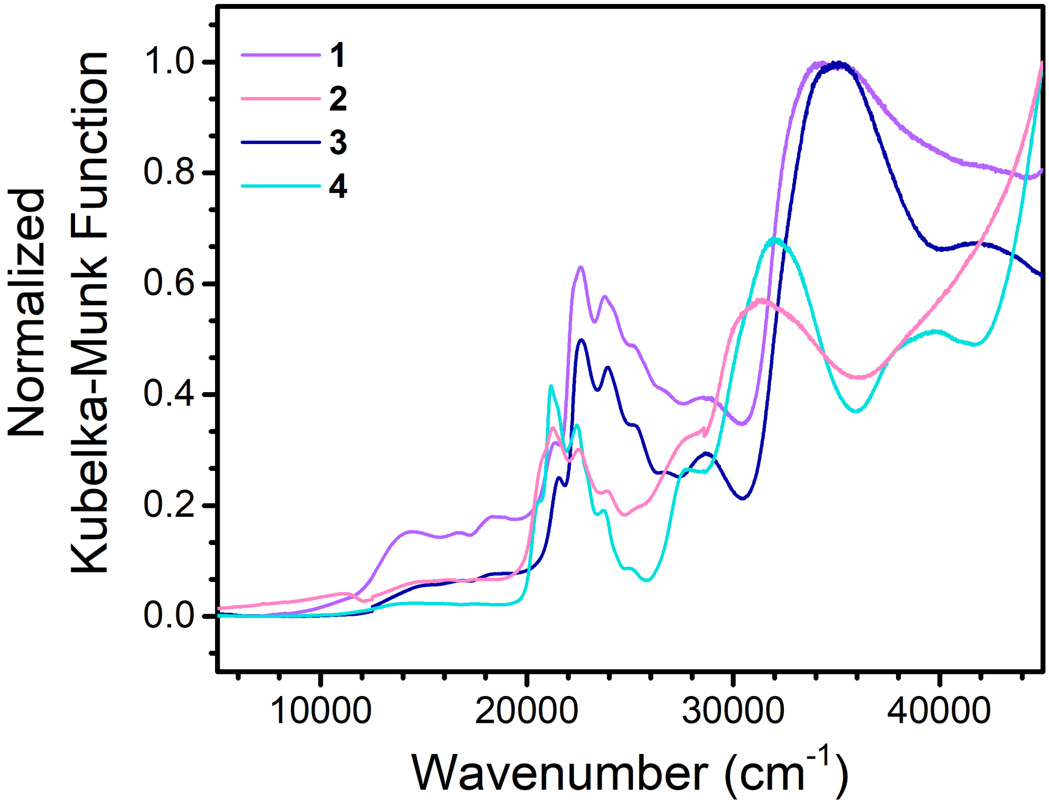

Figure S14 | Diffuse reflectance (UV-Vis) spectra for **1**–**4.** Spectra were collected at room temperature under nitrogen.

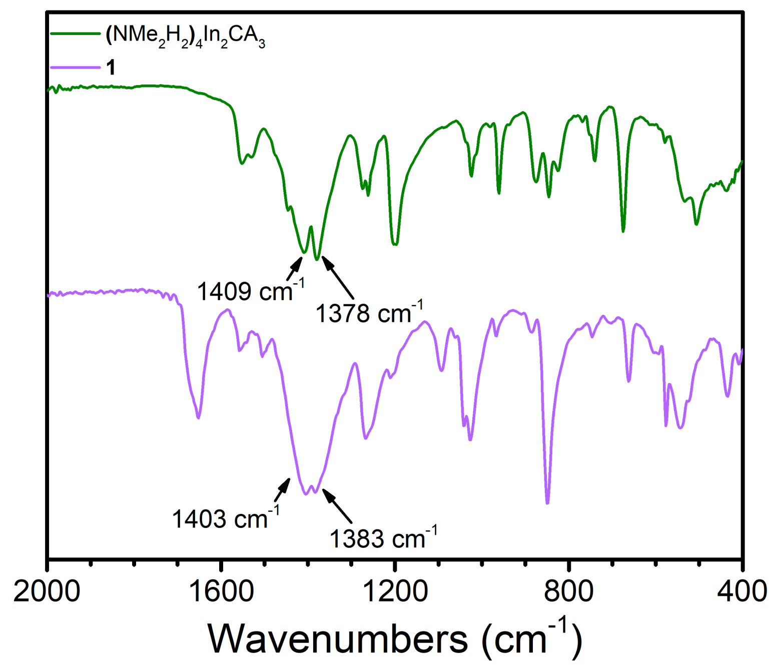

Figure S15 | Infrared spectroscopic data on **1** (purple) and $(NMe_2H_2)_4In_2CA_3$ (green). All data were collected on solid samples at room temperature under a dinitrogen atmosphere.

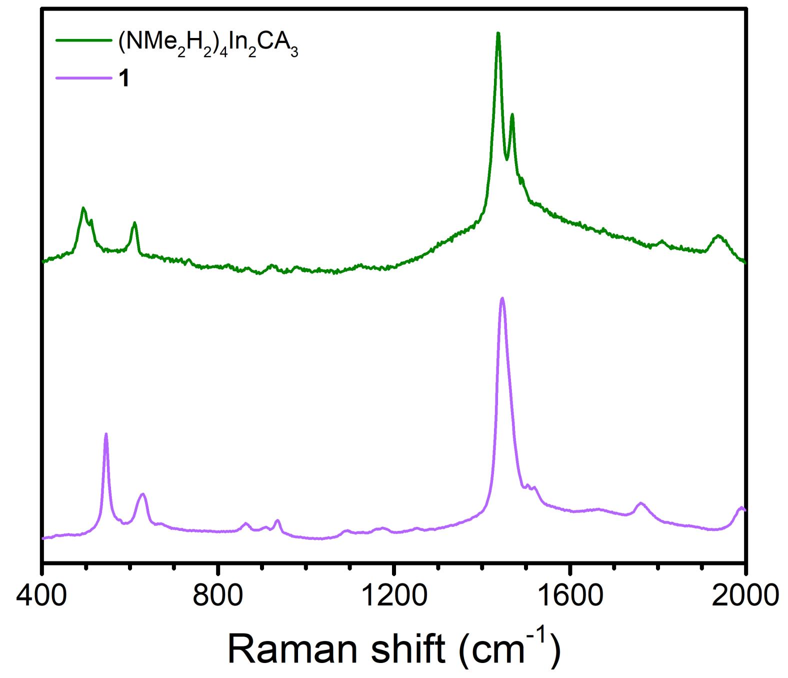

Figure S16 | Resonance Raman spectroscopic data on **1** (purple) and $(NMe_2H_2)_4In_2CA_3$ (green). All data were collected in sealed boron-rich glass capillaries at room temperature with λ_{ex} = 473 nm.

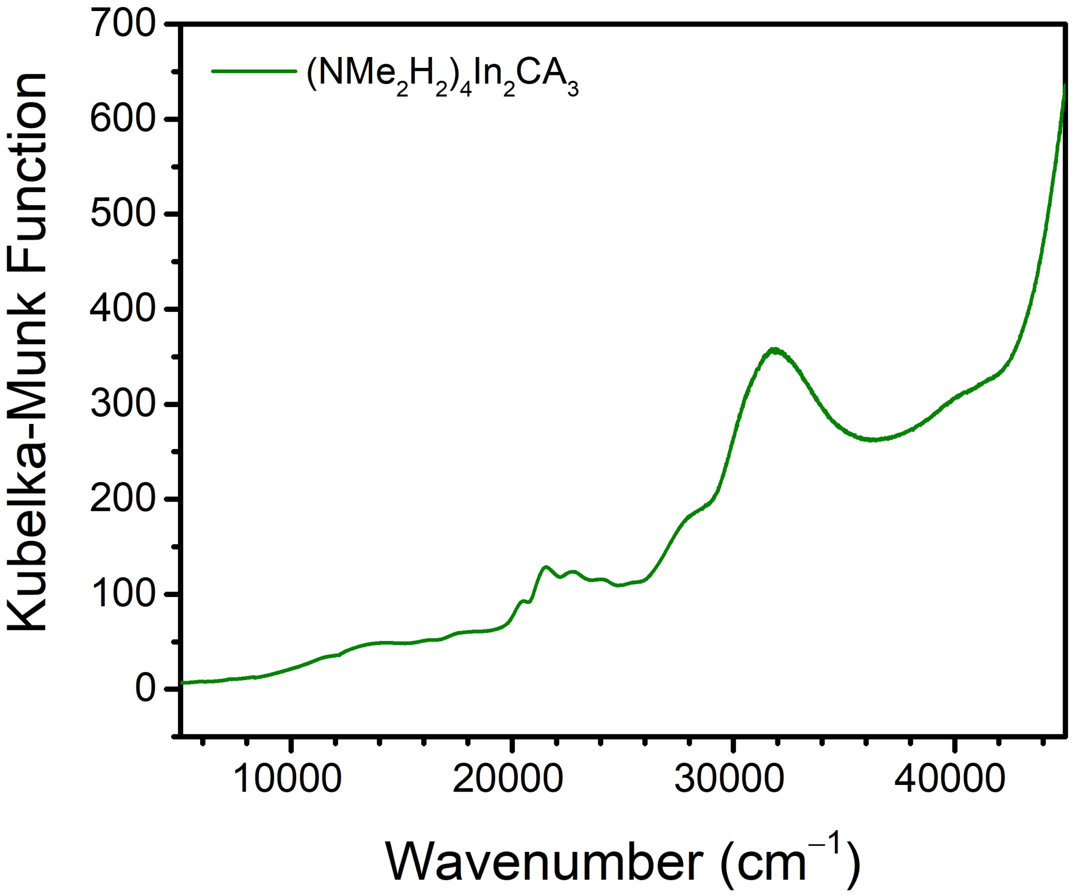

Figure S17 | Diffuse reflectance (UV-Vis) spectrum for $(NMe_2H_2)_4In_2CA_3$. Spectrum was collected at room temperature under nitrogen.

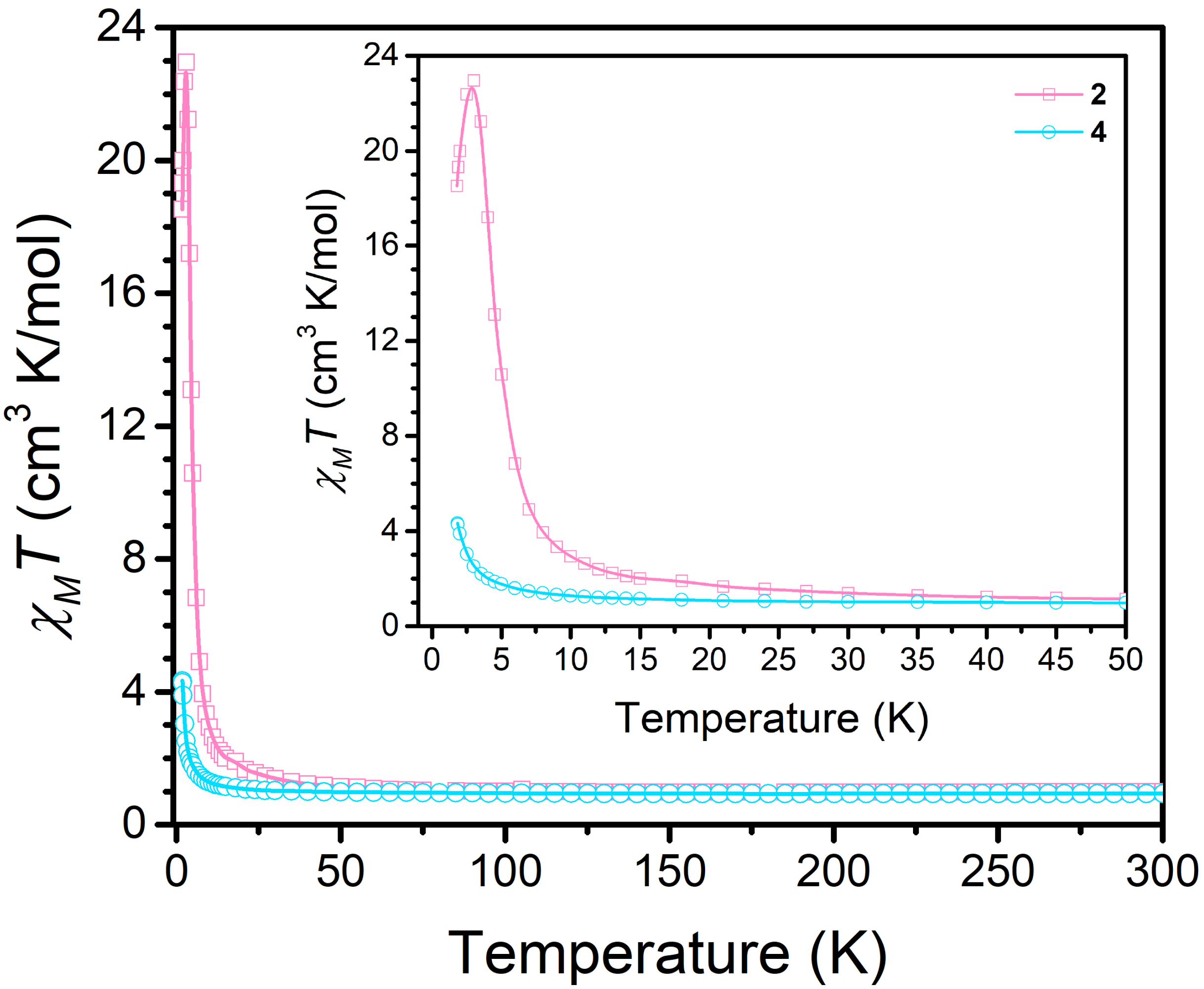

Figure S18 | Dc magnetic susceptibility of **2** and **4** from 1.8 to 300 K at 0.1 T. The inset highlights the difference of the maximum magnetic moment between **2** and **4** at low temperatures. The lines are guides to the eye.

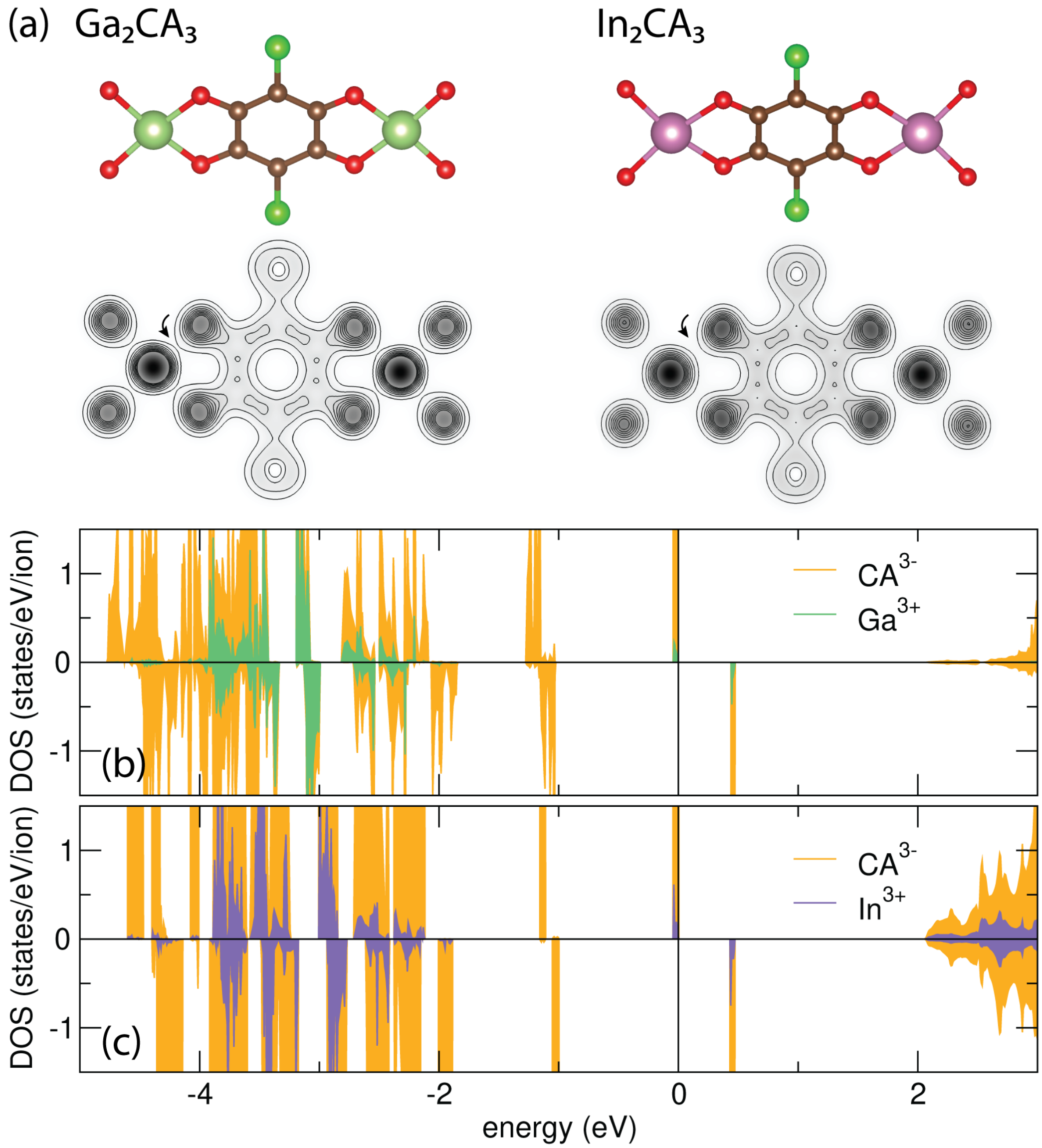

Figure S19 | (a) A portion of the atomic structure of Ga_2CA_3 and In_2Ca_3 viewed down the $[\bar{1}\bar{1}2]$ direction and the corresponding two-dimensional charge density showing enhanced covalency through the asymmetric charge distribution between the bidentate oxygen ligands comprising the chloranilate anion and the chelated Ga cation (left arrow) compared to the In cation (right arrow). Oxygen, carbon, chlorine, gallium, and indium are depicted as red, brown, bright green, and purple spheres, respectively. Projected density-of-states (DOS) of (b) Ga_2CA_3 and (c) In_2CA_3 focusing on the low-energy states formed by the main group metal cations and chloranilate anion.

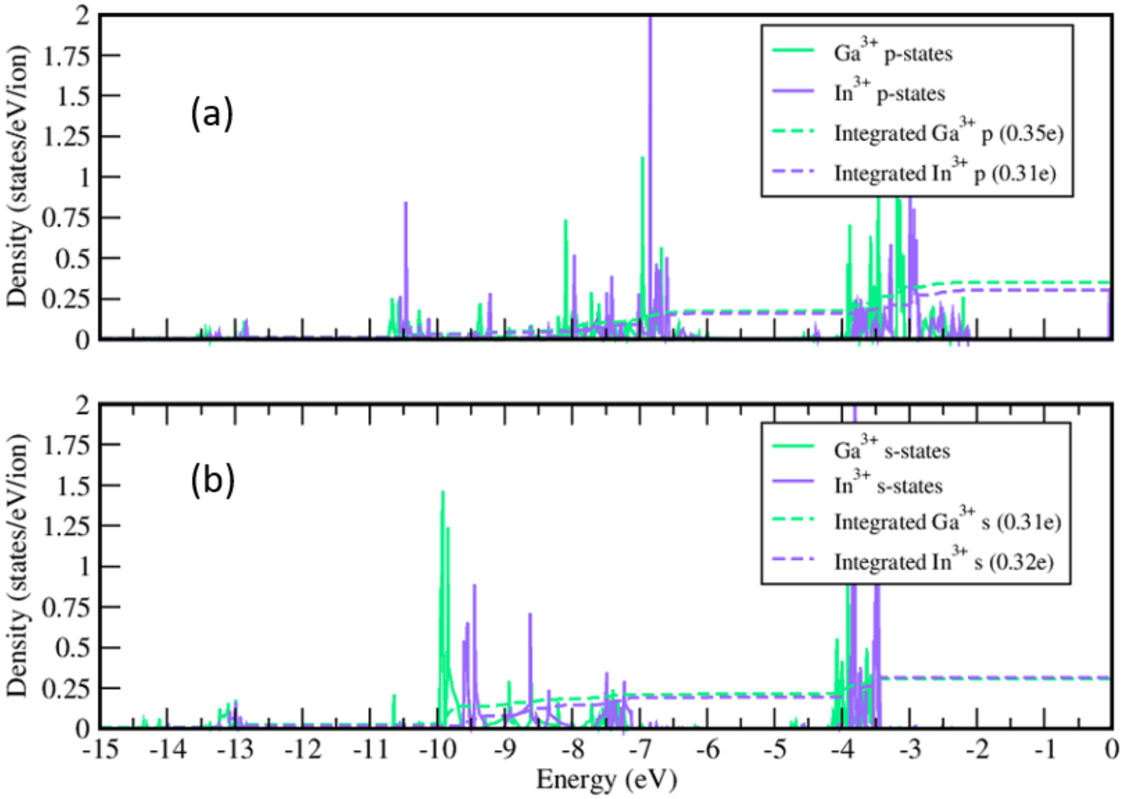

Figure S20 | The projected DOS for Ga^{3+} and In^{3+} were integrated below the Fermi level in order to demonstrate the change in occupancy between both compounds which reflect the degree of hybridization. This is shown by dashed line with Gallium being depicted in bright green while Indium is in purple. The total occupation is shown in parenthesis. (a) The projected DOS of the p-states show that Ga^{3+} has more occupancy thus greater hybridization. (b) The integrated s-states show that Ga^{3+} and In^{3+} have comparable occupancies, so their hybridization would have a lesser contribution to the overall difference in superexchange.

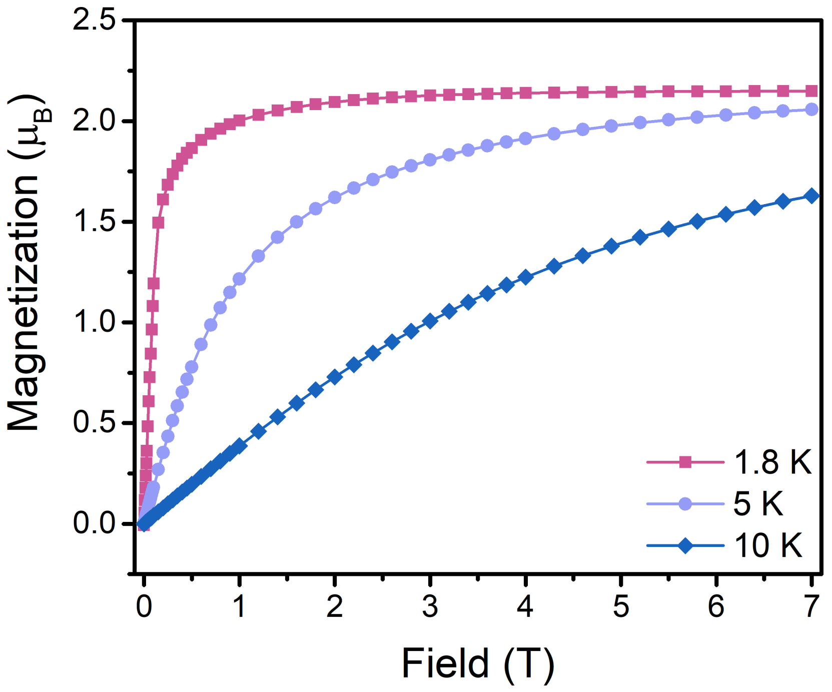

Figure S21 | Variable field magnetization data for **1** at temperatures of 1.8 K, 5.0 K, and 10.0 K from 0 T to 7 T. Lines are guides to the eye.

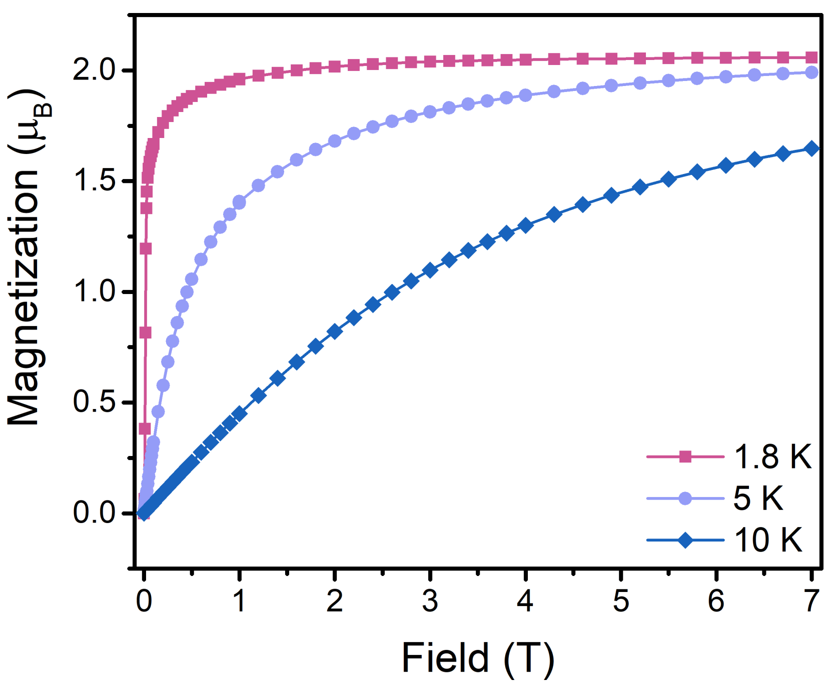

Figure S22 | Variable field magnetization data for **2** at temperatures of 1.8 K, 5.0 K, and 10.0 K from 0 T to 7 T. Lines are guides to the eye.

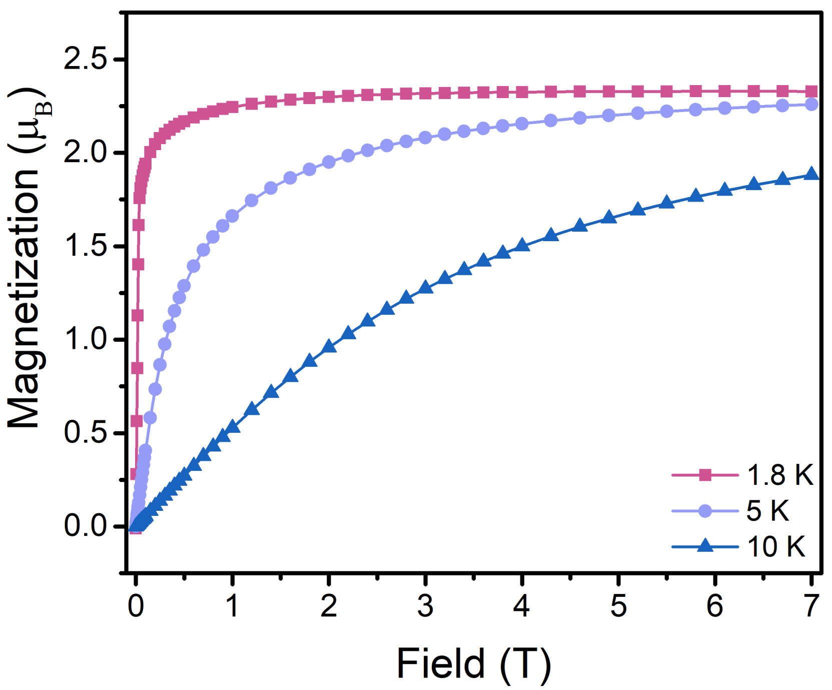

Figure S23 | Variable field magnetization data for **3** at temperatures of 1.8 K, 5.0 K, and 10.0 K from 0 T to 7 T. Lines are guides to the eye.

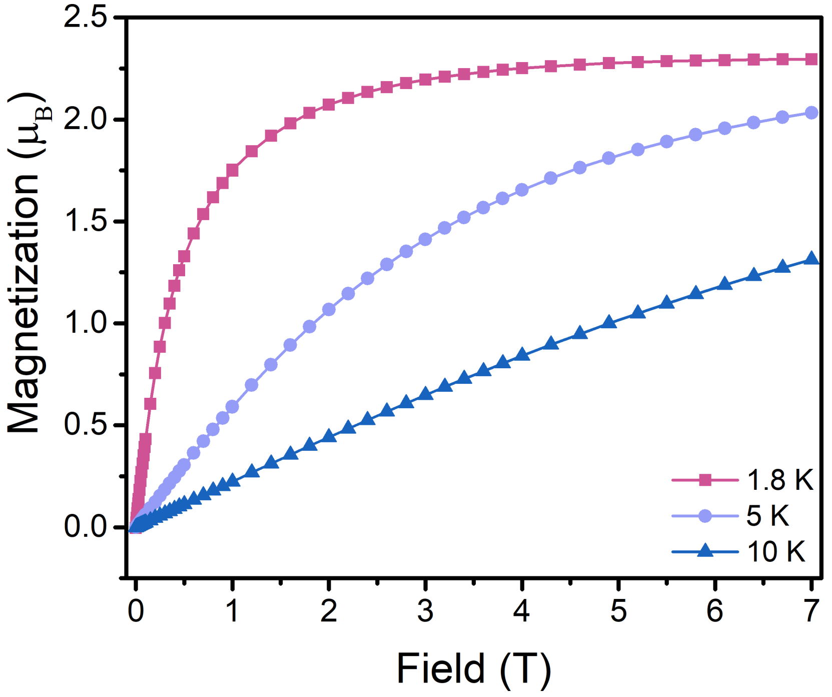

Figure S24 | Variable field magnetization data for **4** at temperatures of 1.8 K, 5.0 K, and 10.0 K from 0 T to 7 T. Lines are guides to the eye.

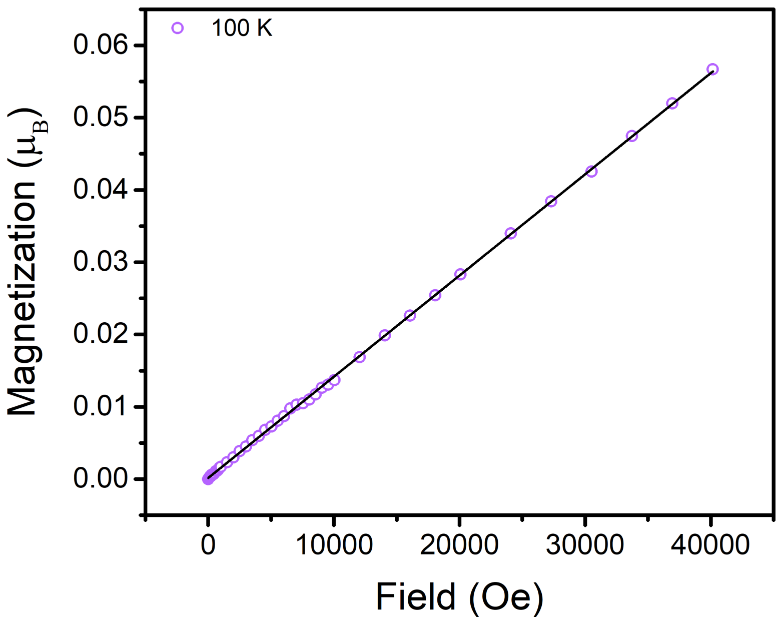

Figure S25 | Variable-field magnetization of a polycrystalline samples of **1** restrained under eicosane acquired at 100 K. The black line is a linear fit to the data illustrating the absence of ferromagnetic impurities.

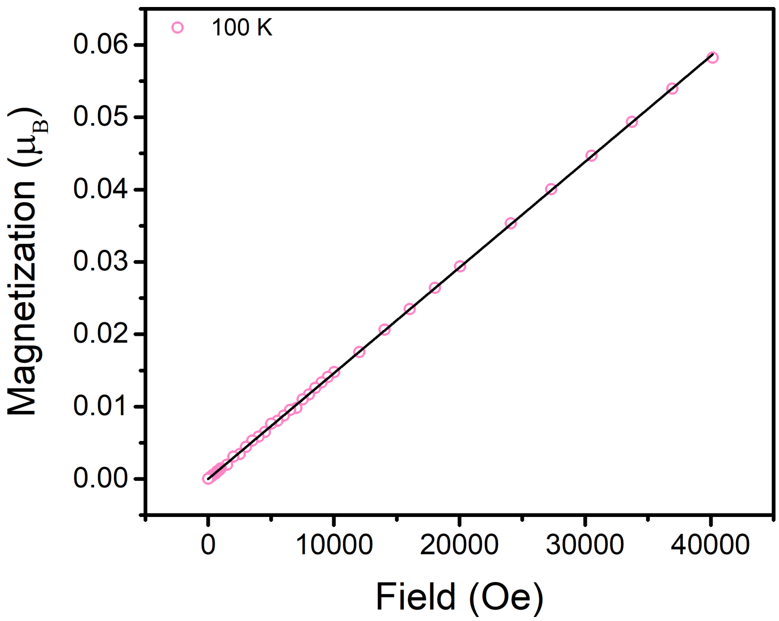

Figure S26 | Variable-field magnetization of a polycrystalline samples of **2** restrained under eicosane acquired at 100 K. The black line is a linear fit to the data illustrating the absence of ferromagnetic impurities.

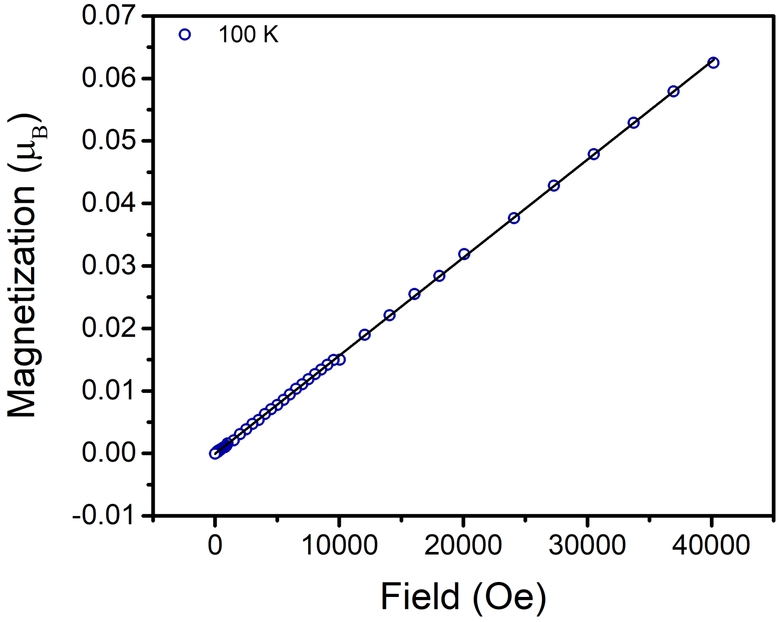

Figure S27 | Variable-field magnetization of a polycrystalline sample of **3** restrained under eicosane acquired at 100 K. The black line is a linear fit to the data illustrating the absence of ferromagnetic impurities.

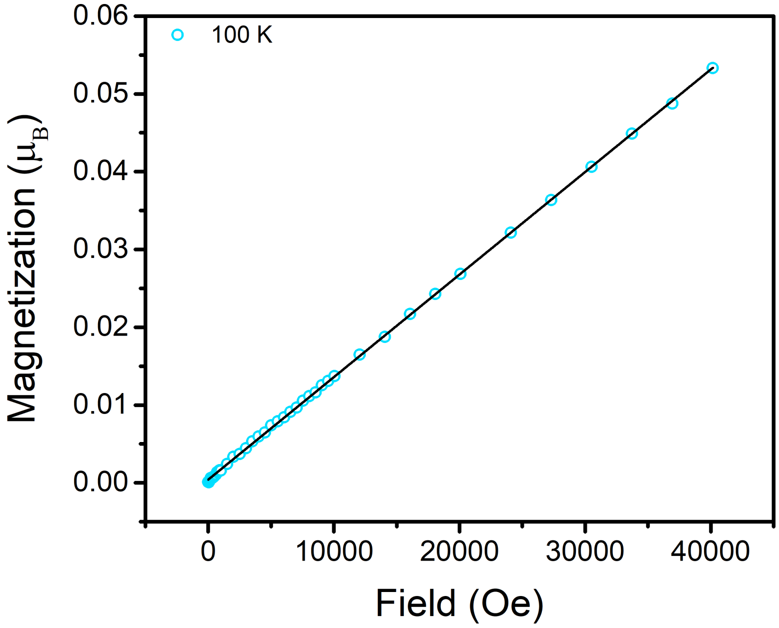

Figure S28 | Variable-field magnetization of a polycrystalline sample of **4** restrained under eicosane acquired at 100 K. The black line is a linear fit to the data illustrating the absence of ferromagnetic impurities.

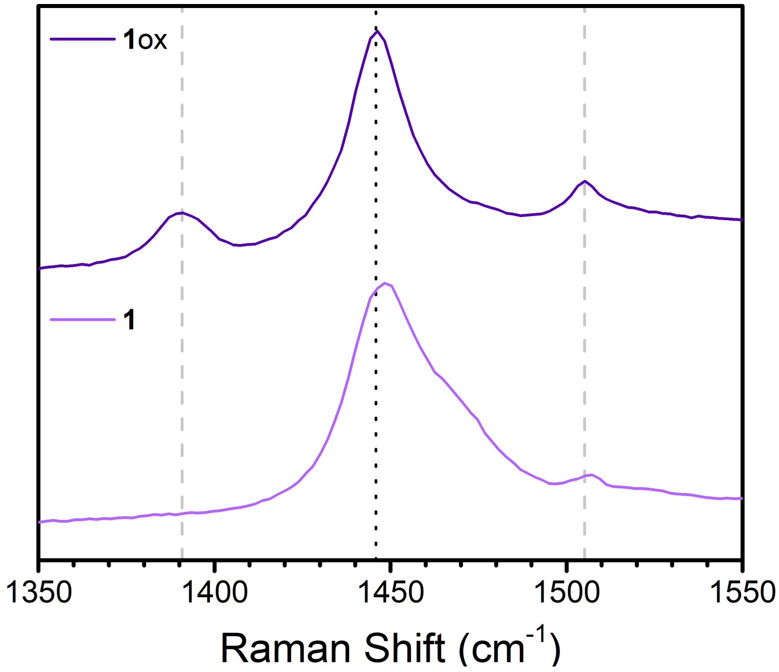

Figure S29 | Raman spectroscopic data on **1** (purple) and **1ox** (dark purple). All data were collected at room temperature on solid samples in boron-rich capillaries sealed with UV curable epoxy under a dinitrogen atmosphere.

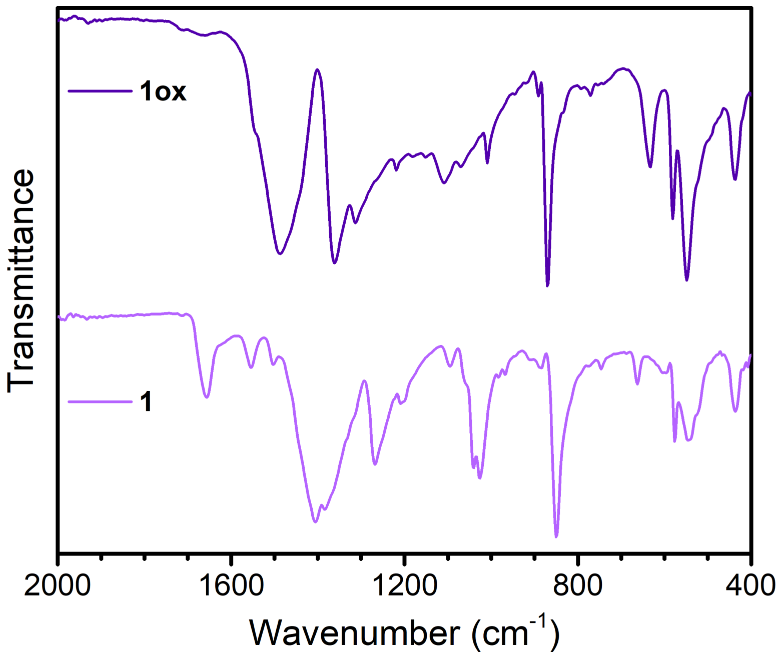

Figure S30 | Infrared spectroscopic data on **1** (purple) and **1ox** (dark purple). All data were collected on solid samples at room temperature under a dinitrogen atmosphere.

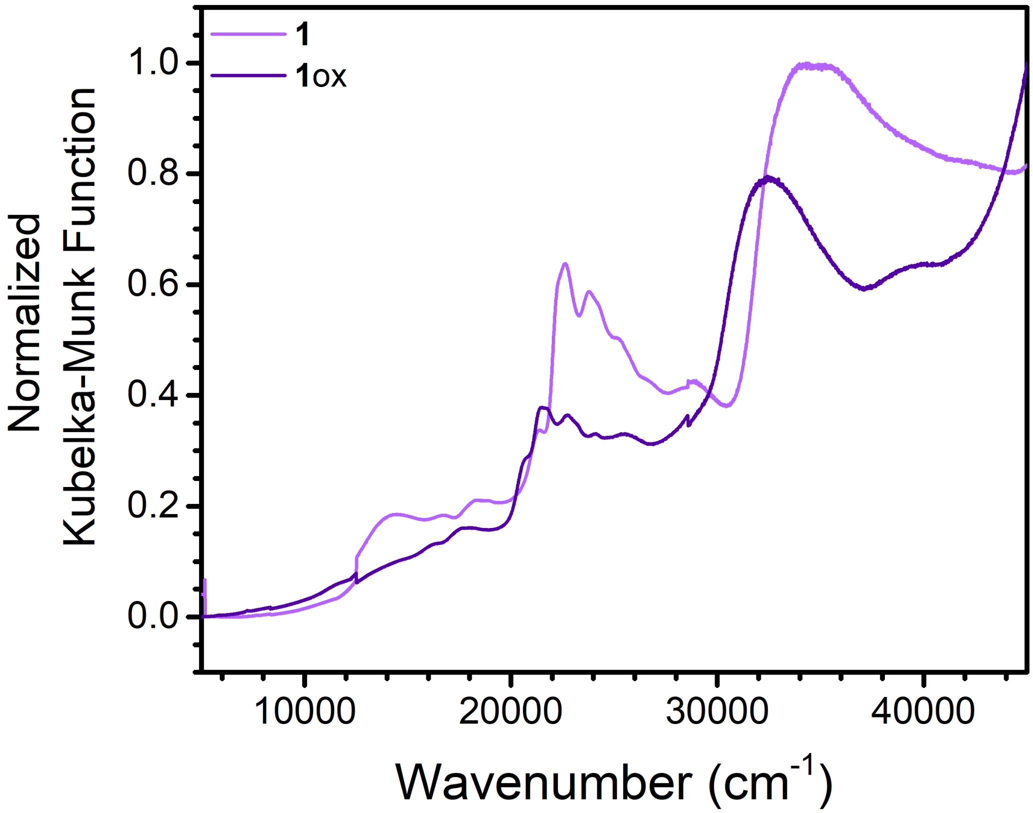

Figure S31 | Diffuse reflectance (UV-Vis) spectra for **1** (purple) and **1ox** (dark purple)**.** Spectra were collected at room temperature under dinitrogen.

Attempting to remove the intercalated ferrocenium ions by cation metathesis with tetraethylammonium salts results in the residual ions oxidizing the framework to an amorphous material containing only CA^{2-} which we confirmed with Raman and FTIR spectroscopies.

References

(1) D. Guo and J. K. McCusker, *Inorg. Chem.*, 2007, **46**, 3257–3274.
(2) Spengler, R.; Lange, J.; Zimmermann, H.; Burzlaff, H.; Veltsistas, P. G.; Karayannis, M. I. *Cryst B* **1995**, *51* (2), 174–177.
(3) Bain, G. A.; Berry, J. F. *J. Chem. Educ.* **2008**, *85* (4), 532.
(4) G. Kresse, Furthmüller, J. *Phys. Rev. B.* 1996, **54***,* 11169.
(5) G. Kresse, D. Joubert *Phys. Rev. B.* 1999, **59**, 1758.
(6) P.E. Blöch, *Phys. Rev. B*. 1994, **50***,* 17953.
(7) J.P. Perdew, A. Ruzsinszky, G.I. Csonka, O.A. Vydrov, G.E. Scuseria, *Phys. Rev. Lett.* 2008, **100**, 136406
(8) H.J. Monkhorst, J.D. Pack, *Phys. Rev. B ,*1976, **13**, 5188–5192
